# Disordered eating behavior and adolescent social health: evidence of interpersonal disruption across time and relationships

**DOI:** 10.1093/abm/kaag029

**Published:** 2026-06-16

**Authors:** Christopher D Otmar, Colbey Ricklefs, Jason M Nagata

**Affiliations:** Department of Pediatrics, University of California, San Francisco, San Francisco, CA 94158, United States; Department of Pediatrics, University of California, San Francisco, San Francisco, CA 94158, United States; Department of Pediatrics, University of California, San Francisco, San Francisco, CA 94158, United States

**Keywords:** disordered eating, social health, adolescent relationships, peer victimization, family conflict, school connectedness

## Abstract

**Background:**

Disordered eating during adolescence is common and can interrupt daily participation in family, peer, and school life. However, prior studies typically treat social relationships as precursors to disordered eating rather than as health outcomes that may deteriorate once symptoms appear.

**Purpose:**

To test whether adolescents with disordered eating symptoms show poorer social health across multiple relational settings, and whether impairment is stronger for multi-symptom and more persistent symptoms and changes over time.

**Methods:**

Data came from 11 868 adolescents in the Adolescent Brain Cognitive Development Study, followed annually across 5 years. Parents reported 5 eating disorder-related symptoms each year; variables were created for any symptom, ≥2 symptoms, and the proportion of study years with symptoms (chronicity). Social health was measured with parent-reported social withdrawal and adolescent-reported prosocial behavior, peer victimization, family conflict, and school functioning (school disengagement, school environment, and school involvement). Generalized estimating equations with year fixed effects estimated population-averaged associations.

**Results:**

Across adolescence, 39.7% of adolescents were ever symptomatic (eg, binge eating, fear of gaining weight, or vomiting for weight control) and 7.0% had ≥2 symptoms. Any eating disorder symptom was associated with higher social withdrawal (β = 0.26, *q* < .001), more peer victimization (βs = 0.09-0.12, *q*s < .001), and greater family conflict (β = 0.12, *q* < .001); school environment and involvement were modestly lower. Associations were substantially stronger for multi-symptom and more persistent symptoms, such that adolescents symptomatic across multiple years showed the largest deficits across all layers of social health. Symptom-by-year tests indicated the social withdrawal gap widened modestly over time.

**Conclusions:**

Adolescent disordered eating symptoms were associated with poorer social health across family, peer, and school settings, particularly when symptoms are recurrent or multifaceted. These findings suggest that social participation warrants assessment alongside eating disorder screening in pediatric and school contexts.

Disordered eating is increasingly prevalent among adolescents and affects nearly 1 in 5 children and adolescents worldwide.^1–3^ Pediatric presentations often disrupt growth, puberty, and daily participation in school, family, and peer life.^4^ These early disruptions can compromise both physical and interpersonal functioning.^5–7^ For many, eating disorder symptoms that begin in adolescence can generate enduring medical and interpersonal consequences that carry into adulthood.^8–11^ Despite this, the social costs of disordered eating behaviors during adolescence remain underexamined, particularly in population-based samples followed across multiple years.

Adolescents’ eating patterns are structured by the social routines of daily life. Shared meals often serve as a key site for family and peer interaction by providing opportunities for belonging and behavioral modeling.^12–14^ When eating becomes restrictive or compulsive, these routines are disrupted and reduce opportunities for supportive engagement and reinforce isolation.^5^^,^^15^ Behaviors commonly associated with disordered eating, such as withdrawal^16^ and irritability,^17^ can strain relationships and erode the social reinforcement needed to sustain healthy behavior. Thus, eating disorder symptoms reflect not only individual distress but also disruptions in the relational processes through which adolescents regulate emotion and maintain interpersonal functioning.

## Social health and eating disorders

The World Health Organization Commission on Social Connection (2025),^18^ alongside a Lancet Public Health editorial,^19^ identified social health as a “neglected third pillar” of well-being, one that is coequal with physical and mental health, and emphasized that connection, participation, and belonging are essential to population health. Building on this framework, Lau et al^20^ propose that social health should be assessed and treated on par with other health dimensions, encompassing the quality of individuals’ relationships, networks, and interpersonal functioning. Together, these perspectives signal a paradigm shift from viewing social relationships solely as determinants of health to recognizing them as intrinsic components of health itself.^21–23^

Despite this conceptual progress, social health remains the least measured and least theorized dimension of adolescent well-being.^24^ Doyle and Link^25^ argue that social health warrants recognition as a legitimate health outcome rather than a mere determinant of physical or psychological status. Dominant health models frequently position relationships as protective factors (ie, buffers against stress or illness) while giving less attention to how psychiatric and behavioral symptoms can erode participation, belonging, and relationship quality.^26^ Reframing social health as an outcome expands the analytic scope of behavioral medicine and clarifies how medical and psychiatric conditions reshape participation in everyday social life.

**Figure 1:**
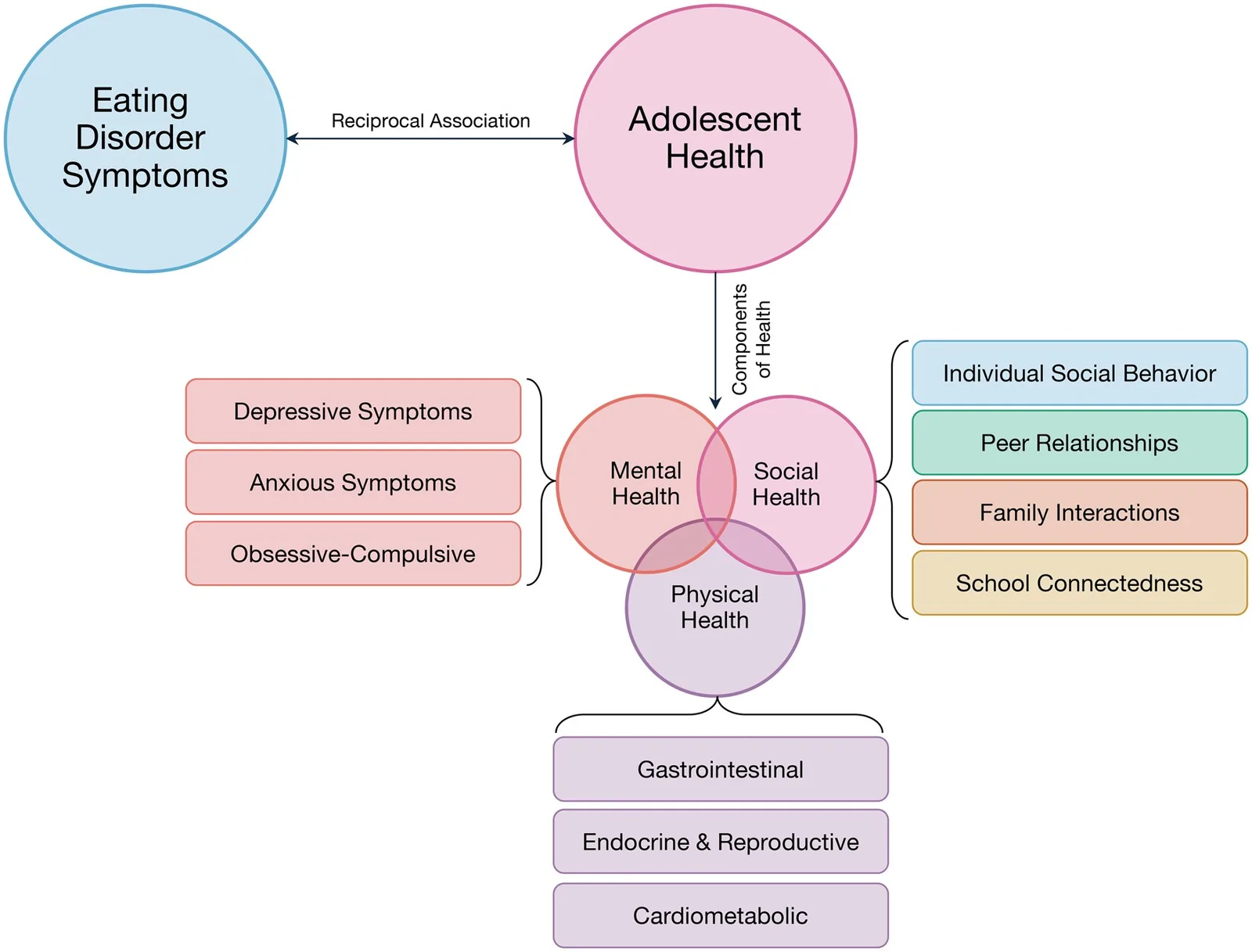
Social-ecological model of adolescent health consequences of eating disorder symptoms. Eating disorder symptoms are positioned as a health condition with physical, mental, and social health. Social health is treated as a distinct component of adolescent well-being, encompassing individual, peer, family, and school contexts. Bidirectional arrows indicate that social impairment may both result from and reinforce eating-related problems.

Extant research has established numerous social antecedents of eating disorder onset, including weight-related teasing^27^^,^^28^ and family conflict.^29^ However, few studies have examined the social consequences of eating disorder symptoms once present. Existing work tends to isolate single relational contexts and rarely assesses whether social difficulties accumulate as symptoms persist over time. The present study addresses these limitations by applying a social-ecological framework^30–32^ to test whether adolescents exhibiting eating disorder symptoms show differences in social health across the self, peers, family, and school (Figure 1), and whether these differences vary by symptom intensity, persistence, and developmental variation across time.

### Layers of social health and eating disorder symptoms

A social-ecological framework conceptualizes adolescents as embedded within interdependent social systems, including individual social behavior, peer relationships, family interaction, and school climate, each contributing uniquely to overall social health.^31^ These layers are active contexts where adolescents learn to regulate emotion, maintain relationships, and establish belonging.^33–35^ Examining these layers in concert provides a coherent way to assess whether eating disorder symptoms are associated with interpersonal disruption across everyday contexts, and whether strain in 1 setting carries over into others over time.

Interpersonal processes have long been central to theoretical models of eating disorders. The cognitive-interpersonal maintenance model of anorexia nervosa describes restrictive symptoms as patterns that can regulate closeness and manage vulnerability in relationships perceived as threatening to autonomy or control.^36^ The interpersonal model of binge eating emphasizes cycles of interpersonal stress, negative self-appraisal, and affect-driven eating, in which episodes may briefly dampen distress after conflict or rejection while increasing isolation and self-criticism.^6^^,^^7^^,^^37–39^ The tripartite influence model further situates these processes within adolescents’ social environments by describing how appearance-related expectations transmitted through parents, peers, and media are internalized and reinforced through comparison processes.^40^ Across these perspectives, a common implication is that eating disorder symptoms can function as interpersonal “reorganizers,” or patterns that alter how adolescents participate in daily relationships. Yet population-based evidence describing how these disruptions present across multiple settings and years remains limited.

### Individual social behavior

The first interpersonal layer concerns adolescents’ direct behavior with others. Self-isolation, or deliberate or protective withdrawal from contact, serves as an affect-regulation strategy when relationships are experienced as demanding or unsafe.^41^ Studies of adolescents with eating disorder symptoms indicate that they often appraise others as unavailable or critical and respond by pulling back, which in turn maintains disconnection.^42^^,^^43^ Elevated rejection sensitivity further predicts avoidance and lowers approach toward peers.^15^ Personality research describes this profile as characterized by low assertiveness, excessive accommodation, and social inhibition.^44–46^

Prosocial behavior, in contrast, reflects cooperative and supportive acts that sustain affiliation.^47–49^ Evidence suggests that adolescents with eating disorder symptoms demonstrate reduced prosocial behavior,^50^ particularly following social exclusion.^51^ Heightened vigilance to evaluation and diminished reward sensitivity orient attention inward, and thus, may constrain spontaneous empathy and cooperative behavior. These findings indicate that eating disorder symptoms not only limit eating behavior but also constrict the relational repertoire required to maintain social bonds. At the same time, others argue that some eating behaviors may paradoxically reflect pathological altruism, or an overextension of care toward perceived social demands at the expense of one’s own well-being.^52^ These patterns may appear contradictory, yet both can be understood as regulatory responses to perceived social threat. Whether an adolescent withdraws from relational demands or overextends care toward others, the underlying function may be the same, serving to manage evaluative concern and preserve social standing.

### Peer relationships

Peer relations constitute a central dimension of adolescent social life. Peer victimization encompasses overt (verbal or physical aggression), reputational (gossip or online disparagement), and relational (exclusion or ostracism) forms of harm.^53–55^ Although these manifestations differ in visibility, each enforces peer norms around appearance, emotional composure, and participation. Adolescents exhibiting eating disorder symptoms may be especially vulnerable to these pressures.^27^^,^^56^ For instance, restrictive eating behaviors can appear moralizing or reserved, whereas binge-purge symptoms often evoke gossip or ridicule.

Longitudinal research shows that bullying can precede eating disorder onset,^57^ but eating-related symptoms can also heighten later victimization risk. Features such as rigidity around food, secrecy, or episodes of loss of control may make social behavior less predictable and elicit negative peer responses. Adolescents high in rejection sensitivity or low in interpersonal trust may also misinterpret neutral social interactions as threatening.^15^^,^^58^ Over time, these processes may consolidate defensive interpersonal patterns that reduce peer support and increase risk for sustained social isolation.

### Family interaction

Families are another context in which adolescents learn norms surrounding nutrition, appetite, and emotional expression.^59–61^ Recurring family conflict within this environment, characterized by the expression of incompatible goals and expectations, can disrupt the coordination of care.^62^ Empirical studies have found that destructive family conflict is linked to poorer overall family functioning.^63^^,^^64^ Because eating disorder symptoms often provoke disagreements over food, weight, and control, they can activate preexisting family tensions or create new ones. Outside a structured family-based treatment context, surveillance around eating may increase conflict rather than resolve it.^65–67^ Communication research has demonstrated that families vary in how openly they discuss differences versus how strongly they enforce conformity.^68^^,^^69^ When conversation is restricted and parental control is high, conflict tends to center on obedience rather than understanding, leaving adolescents with few ways to express distress or negotiate autonomy. When symptoms develop, meal-related interactions often become a focus of tension.

### School connectedness

Schools combine daily peer contact, adult oversight, and institutional norms, making them key environments for adolescent interpersonal functioning.^70^ Yet school connectedness has rarely been examined as an outcome affected by eating disorder symptoms.^71^ Most studies position schools as sites for prevention or screening rather than as relational environments through which health conditions circulate and accumulate effects. Nonetheless, evidence links school climate and perceived belonging to eating disorder symptom expression.^72^^,^^73^ Extending ecological and family-systems perspectives, Winter et al^74^ conceptualize schools as relational ecologies in which educators occupy pivotal roles within the home-school mesosystem. Within these ecologies, the quality of interactions among teachers, parents, and peers can stabilize or destabilize daily functioning. The present framework treats school connectedness as a multidimensional construct encompassing^75^: (1) school environment (feeling safe, supported, and recognized by adults); (2) school involvement (motivation, enjoyment, and classroom participation); and (3) school disengagement (boredom and detachment from institutional goals). Eating-related distress may lower perceived support, reduce engagement, and increase disengagement as energy and confidence decline.

### Symptom intensity, persistence, and developmental variation

**Figure 2:**
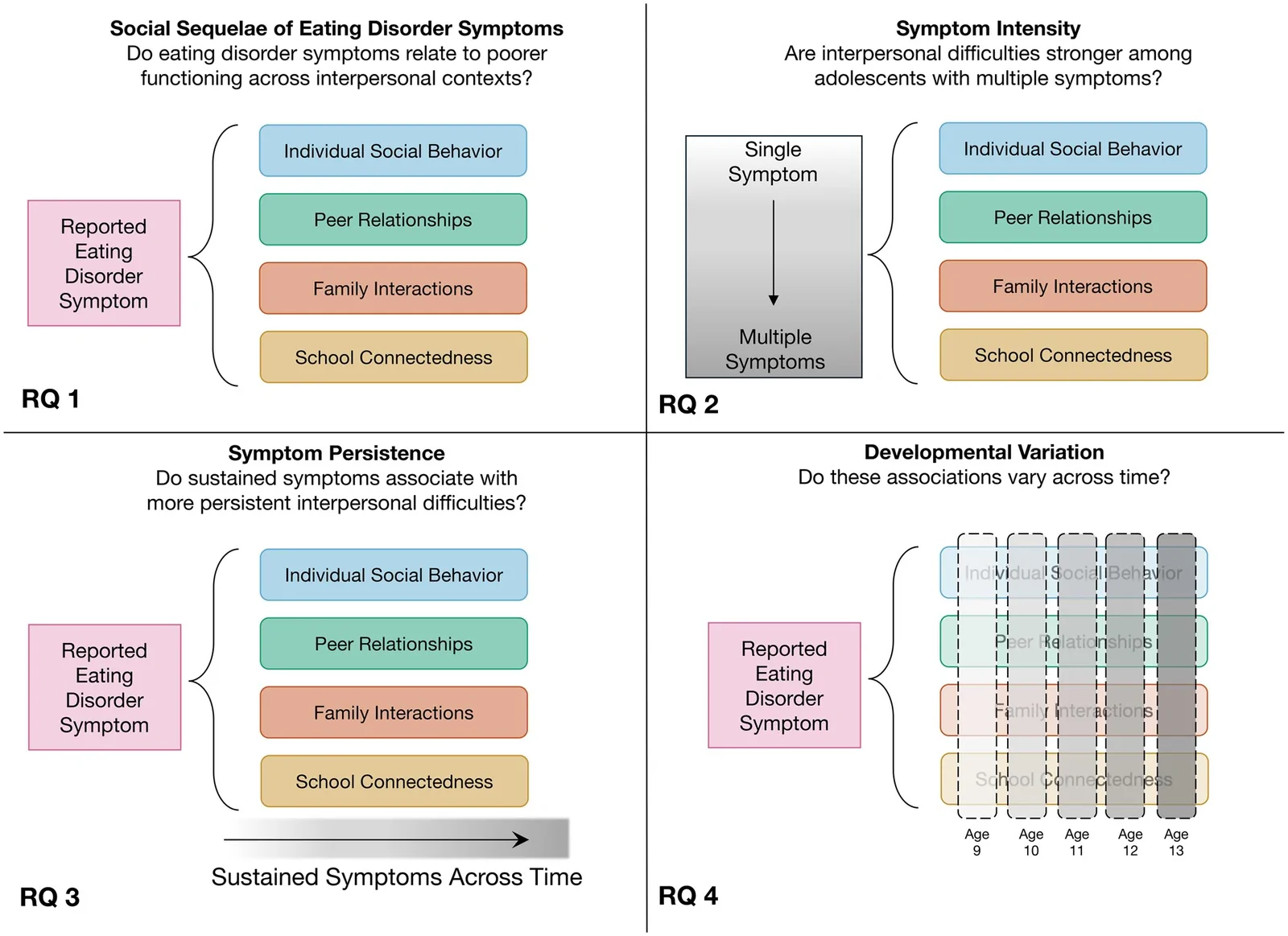
Framework for testing social health sequelae of eating disorder symptoms across adolescence. This figure depicts the conceptual and analytic framework used to test the social health correlates of parent-reported eating disorder symptoms in adolescence. Panel A (Research Question 1) tests whether adolescents with any symptom history (≥1 symptom at any wave) show poorer functioning across individual social behavior, peer relationships, family interactions, and school connectedness, compared with adolescents with no symptoms. Panel B (Research Question 2) tests symptom intensity by examining whether multisymptom histories (≥2 symptoms at any wave) are associated with stronger impairment than the ≥1-symptom specification. Panel C (Research Question 3) tests symptom persistence using proportional exposure indices (fraction of study years meeting the ≥1- or ≥2-symptom criterion; 0-1), interpreted as contrasts between adolescents who were never symptomatic and those symptomatic in every observed year. Panel D (Research Question 4) tests developmental variation by estimating eating disorder symptom-by-study-year interactions and, when jointly significant, year-specific contrasts.

Understanding the social impact of eating disorder symptoms requires attention to time.^76^^,^^77^ Three temporal features can clarify how social effects may become more evident across adolescence: intensity, persistence, and developmental variation.^78^^,^^79^ Intensity refers to how severe and how numerous the eating-related symptoms are. When restrictive eating, purging, or compulsive exercise occur together, the combined physiological and cognitive demands may coincide with greater self-focus and more limited participation in everyday relationships. Persistence refers to how consistently symptoms recur over time. When symptoms persist over longer periods, family and peer routines may gradually adjust to the adolescent’s patterns, and social engagement can become more constrained. Developmental variation refers to whether the strength of symptom related social differences changes over time as adolescents move through early adolescence. During this period of life, even relatively modest disturbances in eating behavior may correspond with changes in emerging patterns of belonging. Considering these temporal features helps describe which adolescents are most likely to show weaker interpersonal functioning and why those with sustained or multifaceted symptoms may require closer attention (Figure 2).

At the same time, disordered eating symptoms vary in observability and likely severity. For example, fear of weight gain may be more common and less behaviorally visible than binge eating or vomiting, and different symptoms may relate to different interpersonal patterns. This symptom heterogeneity is central to interpreting population-based prevalence estimates and to specifying which symptom features track social functioning most consistently.

## The present study

The present study integrates interpersonal and social-ecological perspectives within a developmental framework. Using repeated measures across 5 annual assessments in a national cohort, we address 4 questions that map directly onto intensity, persistence, and developmental variation:

Research Question 1 (overall exposure): Do adolescents who exhibit any eating disorder symptom show poorer social health across individual behavior, peers, family, and school relative to adolescents with no symptoms?

Research Question 2 (intensity): Are associations stronger among adolescents with multiple concurrent symptoms than among adolescents with lower symptom counts?

Research Question 3 (persistence): Are associations stronger among adolescents with symptoms recurring across multiple years than among adolescents with more transient symptom expression?

Research Question 4 (developmental variation): Do associations between eating disorder symptoms and social health differ across study years, indicating changes in strength or direction over time?

Consistent with these questions, we expected that adolescents exhibiting eating disorder symptoms would show higher social withdrawal, greater peer victimization, higher family conflict, and lower school connectedness. We further expected that associations would be patterned by symptom intensity and persistence. Finally, we evaluated whether associations varied across waves and whether specific symptoms were differentially related to social outcomes, given heterogeneity in symptom content and observability.

## Methods

### Participants and procedures

Data were drawn from the Adolescent Brain Cognitive Development (ABCD) Study, a national longitudinal cohort of US adolescents recruited in 2016 and followed annually.^80^^,^^81^ The analytic sample included 11 868 participants with baseline parent-reported KSADS (Kiddie Schedule for Affective Disorders and Schizophrenia) eating-symptom data; wave-specific sample sizes varied across follow-ups due to attrition and item nonresponse. At baseline (year 0), adolescents were 9.96 years old on average (SD = 0.62; range = 8.32-11.28). Sociodemographic characteristics are shown in Table 1. All data were collected under Institutional Review Board approval, and written parental consent and child assent were obtained.

**Table 1:** Sociodemographic characteristics of adolescents at baseline in the ABCD Study (*N* = 11 868; age in years: *M* = 9.96, SD = 0.62, range = 8.32-11.28).

Characteristic		*n* (%)
**Sex**	Female	5676 (47.9)
	Male	6183 (52.1)
**Race/Ethnicity**	Asian	709 (6.0)
	Black	2392 (20.3)
	Latino	2027 (17.2)
	Native American	410 (3.5)
	Other	109 (0.9)
	White	6159 (52.2)
**Parent education**	≤High school	2039 (17.2)
	≥Some college	9792 (82.8)
**Household income**	<$25K	1633 (15.1)
	$25-49K	1588 (14.6)
	$50-74K	1498 (13.8)
	$75-99K	1570 (14.5)
	$100-199K	3310 (30.5)
	≥$200K	1250 (11.5)

Values are unweighted *n* and weighted % at baseline. Percentages are calculated among participants with nonmissing data for each characteristic; therefore, counts within a characteristic may not sum to the full analytic *N* (11 868). Nonmissing *n* (missing *n*) by characteristic: Sex = 11 859 (9); Race/Ethnicity = 11 806 (62); Parent education = 11 831 (37); Household income = 10 849 (1019). Parent education was coded as ≤ high school versus some college or higher (shown as “≥College” in the table). Household income categories refer to annual family income at baseline.

### Measures

#### Eating disorder symptoms

Parents reported annually on 5 symptoms from the Kiddie Schedule for Affective Disorders and Schizophrenia (KSADS-5): binge eating, emaciation/thinness, fear of becoming fat, other weight-control behaviors, and vomiting for weight control. Binary indicators were created for endorsing (1) ≥ 1 symptom and (2) ≥2 symptoms. Proportional indices representing the fraction of study years meeting each criterion captured symptom chronicity. Year-specific prevalence estimates are summarized in Table 2. Although specific validation data for eating disorder symptoms in the KSADS-5 have not been reported, the instrument has demonstrated good convergent validity for other DSM-5 diagnoses (eg, depression, anxiety) and strong parent-adolescent concordance in both interviewer- and self-administered formats.^82^^,^^83^ Prior studies indicate that while parent and child reports of disordered eating often diverge,^84–86^ parent report provides a valid measure of children’s observable eating behaviors, particularly when insight or self-awareness is limited.

**Table 2:** Year-specific prevalence of parent-reported eating disorder symptoms from baseline to year 4.

Year	*n*	≥1 ED symptom, *n* (%)	≥2 ED symptoms, *n* (%)
**0**	11 868	1907 (16.1)	184 (1.6)
**1**	11 219	1896 (16.9)	214 (1.9)
**2**	10 973	1660 (15.1)	195 (1.8)
**3**	10 444	1039 (10.0)	225 (2.2)
**4**	9731	1160 (11.9)	254 (2.6)

Abbreviations: ≥1 ED symptom, endorsement of at least 1 symptom in that year; ≥2 ED symptoms, endorsement of 2 or more symptoms in that year.

Eating disorder (ED) symptoms were assessed annually with parent-reported KSADS-5 items (binge eating, emaciation/thinness, fear of becoming fat, other weight-control behaviors, vomiting for weight control). Percentages reflect the proportion of adolescents with valid ED data in that wave.

#### Social withdrawal

Parents completed the 5-item Child Behavior Checklist (CBCL) detachment dimension.^87^^,^^88^ Items (0-2) were averaged; higher scores reflect greater withdrawal. Internal consistency reliability (Cronbach’s α) increased modestly across waves (α = .64 at year 0; α = .72 at year 4). Item-level withdrawal data were available for most person-waves.

#### Prosocial behavior

Youth completed 3 items from the Strengths and Difficulties Questionnaire Prosocial subscale.^89^ Mean scores (0-2) were computed for helpful and cooperative tendencies. Internal consistency was modest overall and increased across years 0 to 4 (α = .53-.69).

#### Peer victimization

Youth reported overt, relational, and reputational victimization using the Peer Experiences Questionnaire.^55^^,^^90^ Each 3-item subscale was summed (1-5 per item); higher values indicate greater victimization. Peer victimization measures were available beginning at year 2; internal consistency over years 2-4 was acceptable to strong (overt: α = .71-.73; relational: α = .71-.76; reputational: α = .81-.84).

#### Family conflict

Youth completed the 9-item Conflict subscale of the Family Environment Scale^91^ administered via the PhenX protocol.^92^ Items were averaged; higher scores reflect more open hostility and criticism. Internal consistency increased modestly across waves (α = .68 at year 0; α = .72 at year 4).

#### School connectedness

The School Risk and Protective Factors^75^ survey provided subscales for School Environment (eg, feeling safe and supported), School Involvement (eg, liking school), and School Disengagement (eg, boredom). Items were averaged (1-4), with higher scores meaning stronger endorsement. Internal consistency was acceptable for school environment (α = .61-.71) and school involvement (α = .65-.71). School disengagement was a 2-item measure with low internal consistency across waves (α = .19-.31).

Outcome descriptives and missingness by year are summarized in Table S1, and year-specific internal consistency estimates are provided in Table S2.

### Analytic strategy

Population-averaged associations between eating disorder symptoms and each interpersonal outcome were estimated using generalized estimating equations (GEE) and clustering at the participant level.^93–95^ Models included fixed effects for study year (0-4) to adjust for developmental trends. Analyses applied ABCD raked propensity weights to restore representativeness and were adjusted for sex, parent education, and household income. Eating disorder symptoms were modeled in 4 specifications: ever exhibiting 1 or more symptoms, ever exhibiting 2 or more symptoms, and their proportional counterparts captured the fraction of study years meeting each criterion. These proportional indices capture recurrence and chronicity. Primary models used an autoregressive [AR(1)] working correlation, with exchangeable structures producing comparable fit (ΔQIC < 2). False-discovery-rate (FDR) correction was applied across all eating disorder-outcome associations. Both raw *P-*value and FDR-adjusted *q*-values are reported,^96^ with inferences based on the latter. Interactions between eating disorder symptoms and study year tested whether associations varied across adolescence. Wald tests evaluated the joint significance of the interaction terms, and year-specific contrasts were computed when interactions reached significance.

To address symptom heterogeneity, additional models replaced aggregate eating-disorder exposure with each of the 5 KSADS symptoms in turn, retaining the same adjustment set and FDR control across symptom-outcome tests. To address temporality, lagged autoregressive models tested whether eating-disorder symptoms at time *t* − 1 predicted social functioning at time *t* while adjusting for the same outcome at time *t* − 1, year, sociodemographics, and weights.

Finally, to evaluate robustness to developmentally salient covariates and internalizing symptoms, we re-estimated the AR(1) GEE models after adding 3 time-aligned, visit-specific covariates to the base adjustment set: visit age (in years at the assessment), pubertal development (Pubertal Development Scale mean score from that same assessment), and CBCL internalizing *T*-score from that same assessment. These variables were merged by participant and assessment wave, so the covariate value corresponded to the same yearly measurement occasion as the outcome and eating-symptom exposure. Because internalizing adjustment overlaps conceptually (and in our case, empirically) with withdrawal, social withdrawal models were excluded from this sensitivity set. All analyses were conducted in R version 4.5.1 (R Core Team, 2025).^97^

## Results

Across the 5 study waves, 11 868 adolescents contributed data. Nearly 2 in 5 adolescents (39.7%; *n* = 4709) exhibited at least 1 eating disorder symptom during the study, and 7.0% (*n* = 826) endorsed 2 or more. Year-specific estimates showed that roughly 16% of parents reported at least 1 eating disorder symptom for their adolescent at baseline, year 1, and year 2, followed by a decline to 10%-12% at years 3 and 4. Multisymptom presentations were less common, ranging from 1.6% at baseline to 2.6% at year 4. These symptom histories are not mutually exclusive, because the same adolescent-parent dyad could endorse multiple symptoms across waves.

Across years 0-4, emaciation/thinness (16.9%) and binge eating (14.8%) were the most commonly endorsed symptom histories, followed by other weight-control behaviors (13.8%), fear of becoming fat (5.0%), and vomiting for weight control (2.2%) (Table S3). Symptom prevalence shifted across waves in a developmentally patterned way, such that emaciation/thinness was front-loaded and declined sharply by year 3, whereas fear of becoming fat and other weight-control behaviors rose over follow-up; vomiting remained consistently rare (Table S4).

### Overall exposure (Research Question 1)

Adolescents with any eating disorder symptom history (at least 1 symptom at any wave) showed poorer interpersonal functioning across individual, peer, family, and school settings. Parent-reported social withdrawal was higher among adolescents with symptoms (*M* = 0.20) than among those without (*M* = 0.13; *P* < .001, Δ = 0.07). Comparable unadjusted differences were observed for overt, relational, and reputational victimization (Δ = 0.11-0.19, all *P*s < .001) and for family conflict (Δ = 0.03, *P* < .001). Differences in prosocial behavior and school functioning were smaller: the prosocial difference (Δ = −0.01) reached *P* < .001 in the unadjusted model but did not persist once FDR correction and covariate adjustment were applied; school environment and school involvement were modestly lower among adolescents with eating disorder symptoms (Δ = −0.04 to −0.06, *P*s < .001). Overall, adolescents with any eating disorder symptom displayed a broad but modest pattern of interpersonal disadvantage (Table 3).

**Table 3:** Unadjusted social outcomes by lifetime eating disorder symptom history (≥1 symptom at any wave vs never).

Outcome	No ED (0)	Any ED (≥1)	*P*	Δ
**Social withdrawal**	0.13	0.20	<.001	0.07
**Prosocial behavior**	1.69	1.68	<.001	−0.01
**Overt victimization**	3.54	3.65	<.001	0.11
**Relational victimization**	4.58	4.72	<.001	0.13
**Reputational victimization**	3.88	4.07	<.001	0.19
**Family conflict**	0.22	0.24	<.001	0.03
**School disengagement**	2.01	2.05	<.001	0.04
**School environment**	3.30	3.26	<.001	−0.04
**School involvement**	3.21	3.15	<.001	−0.06

Abbreviations: ED, eating disorder; Δ = mean difference (Any ED − No ED); higher scores = more social withdrawal, peer victimization, family conflict, and school disengagement; lower scores = weaker school environment and school involvement.

Peer victimization outcomes are averaged across years 2-4; other outcomes are averaged across years 0-4, as available. *P* values are from GEEs with identity link and clustering on participant to account for repeated measures.

### Symptom intensity and persistence (Research Questions 2 and 3)

Model-based estimates paralleled these descriptive findings and clarified how associations differed by symptom intensity and persistence (Table 4). Across specifications, eating disorder symptoms were positively associated with social withdrawal (β = 0.26, *q* < .001). Stronger effects were observed for multisymptom presentations (β = 0.44, *q* < .001), and proportional exposure, which was the fraction of study years in which symptoms were present (0-1), yielded the largest coefficients (β = 0.78-1.50, *q* < .001). All coefficients are standardized. These proportional-exposure estimates compare adolescents who were never symptomatic to adolescents who were symptomatic in every observed year, so larger values should be interpreted as chronic and recurrent involvement rather than single-wave effects. Prosocial behavior showed no meaningful association with eating disorder symptoms after FDR correction.

**Table 4:** Generalized estimating equation results for eating disorder symptoms predicting social outcomes across adolescence.

Outcome	ED model	β (Exch)	SE (Exch)	β (AR(1))	SE (AR(1))	*p* (AR(1))	*q* (AR(1))
**Social withdrawal**	≥1 ED	0.27	0.02	0.26	0.02	<.001	<.001
	≥1 ED (prop)	0.80	0.05	0.78	0.05	<.001	<.001
	ED ≥ 2	0.46	0.05	0.44	0.05	<.001	<.001
	ED ≥ 2 (prop)	1.55	0.15	1.5	0.15	<.001	<.001
**Prosocial behavior**	≥1 ED	−0.02	0.02	−0.01	0.02	.36	.41
	≥1 ED (prop)	−0.02	0.04	−0.01	0.04	.88	.91
	ED ≥ 2	0.01	0.03	0.01	0.03	.74	.78
	ED ≥ 2 (prop)	0.00	0.09	0.01	0.10	.92	.92
**Overt victimization**	≥1 ED	0.08	0.02	0.09	0.02	<.001	<.001
	≥1 ED (prop)	0.27	0.04	0.27	0.04	<.001	<.001
	ED ≥ 2	0.17	0.04	0.17	0.04	<.001	<.001
	ED ≥ 2 (prop)	0.62	0.13	0.62	0.13	<.001	<.001
**Rel. victimization**	≥1 ED	0.10	0.02	0.10	0.02	<.001	<.001
	≥1 ED (prop)	0.29	0.05	0.29	0.05	<.001	<.001
	ED ≥ 2	0.18	0.04	0.18	0.04	<.001	<.001
	ED ≥ 2 (prop)	0.63	0.14	0.63	0.15	<.001	<.001
**Rep. victimization**	≥1 ED	0.12	0.02	0.12	0.02	<.001	<.001
	≥1 ED (prop)	0.37	0.05	0.37	0.05	<.001	<.001
	ED ≥ 2	0.26	0.05	0.25	0.05	<.001	<.001
	ED ≥ 2 (prop)	0.90	0.17	0.88	0.17	<.001	<.001
**Family conflict**	≥1 ED	0.11	0.02	0.12	0.02	<.001	<.001
	≥1 ED (prop)	0.27	0.04	0.27	0.04	<.001	<.001
	ED ≥ 2	0.13	0.03	0.13	0.03	<.001	<.001
	ED ≥ 2 (prop)	0.37	0.10	0.38	0.10	<.001	<.001
**School disengagement**	≥1 ED	0.04	0.02	0.03	0.02	.06	.07
	≥1 ED (prop)	0.07	0.03	0.06	0.03	.08	.11
	ED ≥ 2	−0.02	0.03	−0.03	0.03	.24	.29
	ED ≥ 2 (prop)	−0.03	0.09	−0.07	0.09	.42	.46
**School environment**	≥1 ED	−0.09	0.02	−0.08	0.02	<.001	<.001
	≥1 ED (prop)	−0.18	0.04	−0.17	0.04	<.001	<.001
	ED ≥ 2	−0.09	0.03	−0.08	0.03	.01	.01
	ED ≥ 2 (prop)	−0.25	0.10	−0.25	0.10	.01	.01
**School involvement**	≥1 ED	−0.08	0.02	−0.07	0.02	<.001	<.001
	≥1 ED (prop)	−0.17	0.04	−0.15	0.04	<.001	<.001
	ED ≥ 2	−0.04	0.03	−0.04	0.03	.25	.29
	ED ≥ 2 (prop)	−0.16	0.10	−0.15	0.09	.13	.16

Abbreviations: ED, eating disorder; Exch, exchangeable working correlation; AR1, autoregressive working correlation.

Population-averaged GEEs with Gaussian family, robust (sandwich) SEs, clustering on participant, and fixed effects for study year (0-4). All models adjust for sex, parent education, and household income and apply ABCD raked propensity weights. ED symptoms were modeled 4 ways: (1) ≥1 ED symptom (occurrence), (2) ≥1 ED symptom, proportion of years (recurrence/chronicity), (3) ≥2 ED symptoms (greater symptom complexity), and (4) ≥2 ED symptoms, proportion of years (persistent, multisymptom exposure). Coefficients (β) are standardized. *q* values are Benjamini-Hochberg FDR-adjusted across all ED-outcome associations and should be used for inference.

Peer outcomes showed the same graded structure. Adolescents with eating disorder symptoms reported more overt, relational, and reputational victimization (β = 0.09-0.12, *q*s < .001). Effects were larger for multi-symptom and proportional symptoms (β = 0.17-0.88, *q*s < .001). Although the models contrasted at least 1 symptom with at least 2 symptoms, rather than exactly 1 symptom with 2 or more, the stepwise increase from occurrence to multi-symptom to proportional exposure is consistent with stronger associations at higher symptom burden and with greater recurrence.

Family conflict followed a similar pattern. Youth with any eating disorder symptom reported higher conflict (β = 0.12, *q* < .001) and multi-symptom cases showed comparable effects (β = 0.13, *q* < .001). Proportional exposure produced larger coefficients (β = 0.27-0.38, *q*s < .001), indicating that recurrent involvement was associated with greater strain beyond the association observed for any lifetime symptom occurrence during the study window (Table 4).

School functioning was weaker and later emerging. Adolescents with eating disorder symptoms reported slightly greater school disengagement, but this association did not survive FDR correction (β = 0.03-0.06, *q*s > .07). Clearer evidence emerged for school environment and school involvement: adolescents with eating disorder symptoms perceived their school climate as less supportive (β = −0.08 to −0.25, *q*s ≤ .01) and reported lower participation and motivation (β = −0.07 to −0.15, *q*s ≤ .001). These associations were strongest in the proportional models, suggesting that sustained exposure to eating disorder symptoms was the context in which school-related differences became most visible. Taken together, these findings offered partial support for the expectation of poorer school connectedness, such that the presence of symptoms was sufficient to produce small differences, but recurrent exposure was the clearer predictor of poorer school environment and involvement (Table 4).

### Developmental variation (Research Question 4)

**Figure 3:**
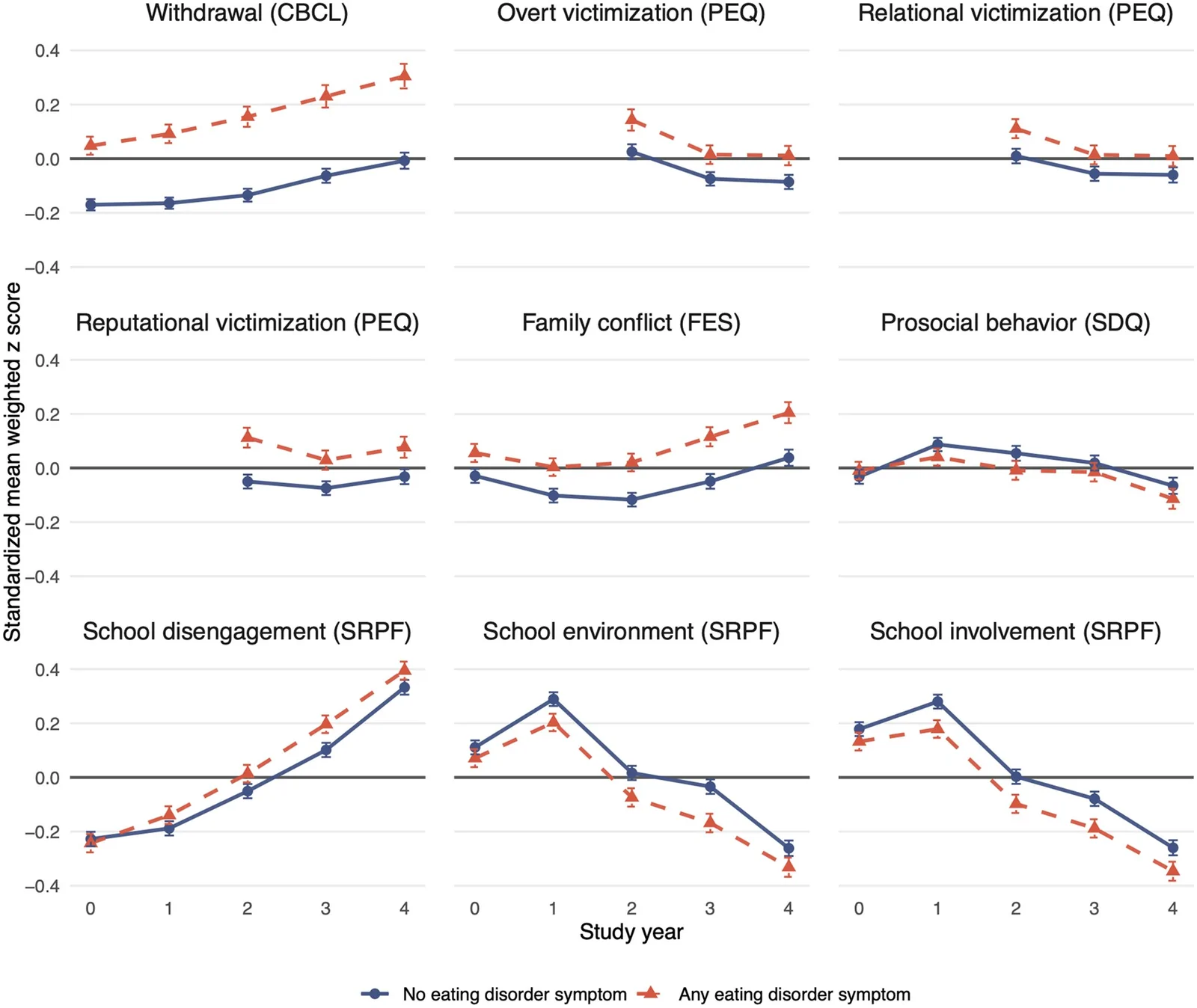
Weighted standardized outcomes over time by eating disorder symptom history (any symptom). This figure plots weighted standardized mean levels of each social outcome across the 5 ABCD assessment waves (study years 0-4), separately for adolescents with no eating-disorder (ED) symptom history versus those with any ED symptom history (ie, parent endorsement of ≥1 KSADS eating-symptom item at any wave). The *x*-axis is study year; the *y*-axis is the standardized mean (weighted *z* score) for each outcome. This figure is a visualization of the developmental variation (ED × year) question using the “any symptom” exposure specification (ie, the same logic as the ED-by-year interaction tests and year-specific contrasts reported for Research Question 4). Formal tests of developmental variation, which were significant for a subset of outcomes only, are reported in Tables S5 and S6.

Eating disorder symptom-by-year interactions tested whether associations differed across study years (Table S5). Year-specific GEE contrasts showed that time variation was concentrated in a subset of outcomes (Table S6). For social withdrawal, effects were present at baseline and increased modestly over time (Table S5). This pattern is shown descriptively in Figure 3. For family conflict, point estimates increased across waves, but the ED × year interaction did not survive FDR correction. Peer victimization effects were relatively stable across years 2-4 (the waves in which victimization was assessed), implying that peer-related differences were established by the first measurement of victimization and then maintained. School-related outcomes showed a more mixed pattern. For the 2-symptom specification, school disengagement varied across study years, shifting from negative at baseline (β = −0.16) to small positive by year 3 (β = 0.07, *q* = .02). By contrast, school environment and school involvement showed some variation in year-specific estimates, but their joint ED × year tests did not survive FDR correction, so these fluctuations should be interpreted cautiously. Prosocial behavior showed limited evidence of temporal variation overall, although the proportional ≥1 symptom specification did show a significant joint interaction.

### Symptom heterogeneity

**Figure 4:**
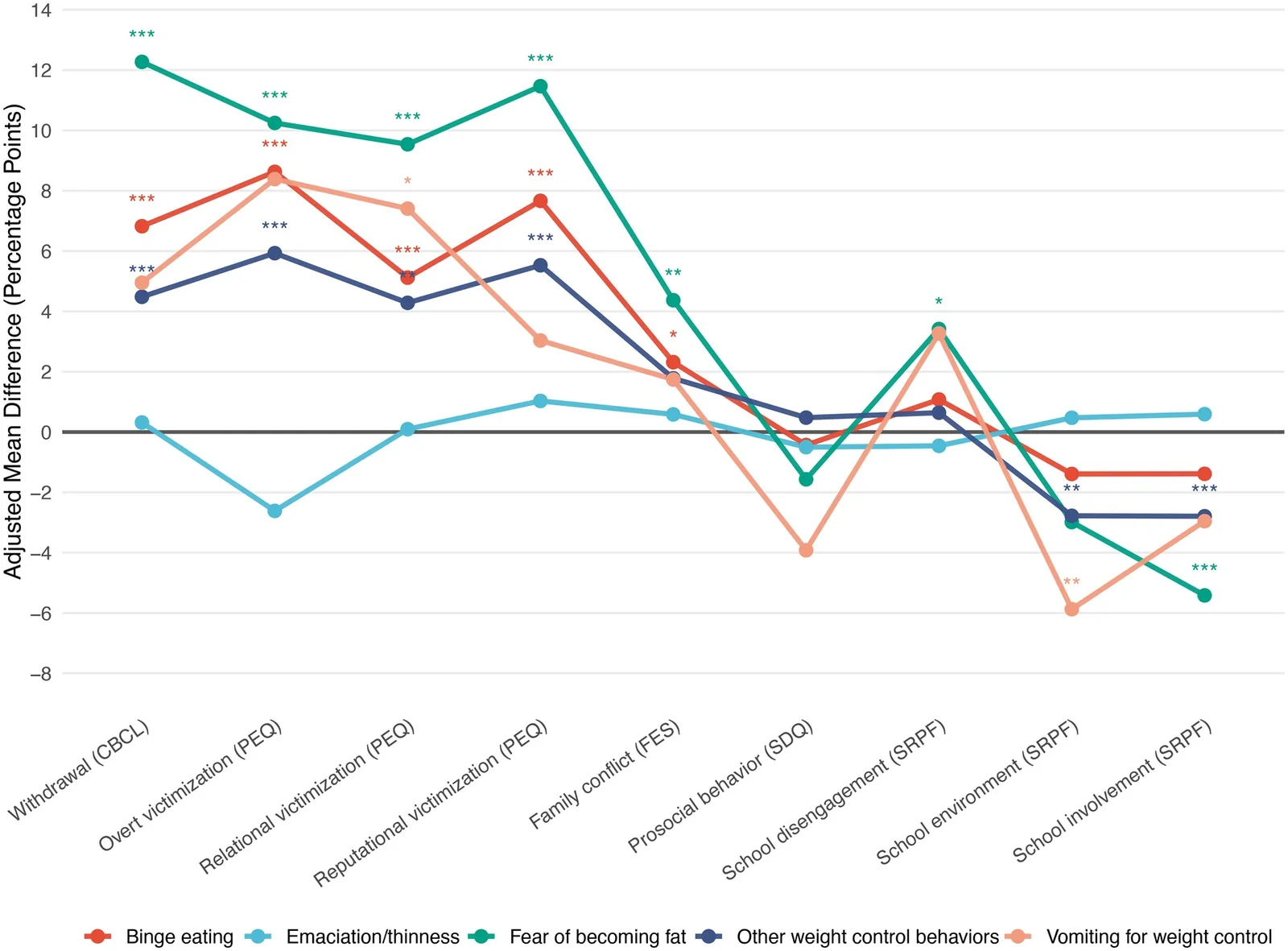
Symptom-specific adjusted associations between disordered eating symptoms and adolescent social health. This figure summarizes symptom heterogeneity of adjusted associations between each specific KSADS eating-disorder symptom (binge eating; emaciation/thinness; fear of becoming fat; other weight-control behaviors; vomiting for weight control) and each social outcome spanning individual, peer, family, and school functioning. The *y*-axis shows the adjusted mean difference (percentage points) for each symptom-outcome pairing, where values above zero indicate higher levels of the outcome among adolescents who ever endorsed that symptom (relative to those who never endorsed it), and values below zero indicate lower levels. Stars mark associations that remain statistically reliable after false discovery rate (FDR) correction (*q* < .05) across symptoms within a given outcome. This figure corresponds to the symptom-specific GEE models used for the symptom heterogeneity analyses (the results summarized in Table S7).

To evaluate whether the aggregate associations were concentrated in particular symptoms, symptom-specific models replaced aggregate exposure with each of the 5 KSADS symptoms in turn (Table S7). Across outcomes, the most consistent symptom correlates of poorer social functioning were binge eating and fear of becoming fat, whereas emaciation/thinness showed limited adjusted associations, and vomiting showed selective associations despite low prevalence. Figure 4 summarizes these symptom-specific adjusted associations across outcomes.

### Prospective models (temporality)

Lagged autoregressive models tested whether eating disorder symptoms at time *t* − 1 predicted social functioning at time t while controlling for the prior level of the same outcome (Table S8). Lagged ≥1-symptom exposure predicted higher subsequent withdrawal (β = 0.09, *q* < .001), higher family conflict (β = 0.06, *q* < .001), higher relational and reputational victimization (βs = 0.06, *q*s = .040), higher school disengagement (β = 0.03, *q* = .045), and lower school environment and involvement (βs = −0.05 and −0.03, *q*s ≤ .040). In contrast, lagged ≥ 2-symptom exposure was largely nonsignificant after correction, with the exception of family conflict (β = 0.10, *q* = .040). Accordingly, statements about prospective prediction are based on these lagged models, whereas pooled GEE and interaction models should be interpreted as population-averaged associations.

### Sensitivity models

Sensitivity models adding time-aligned visit age, pubertal development, and same-wave CBCL internalizing *T*-scores are reported in Table S9.

## Discussion

This study provides one of the largest longitudinal investigations of how eating disorder symptoms relate to adolescents’ interpersonal functioning. Using repeated measures from more than 11 000 US adolescents in a population-based cohort,^80^^,^^81^ we found that parent-observed disordered eating behaviors were consistently associated with modest but pervasive differences in social health. Interpretation of findings, theoretical implications and future directions for this line of research are discussed below.

Consistent with cumulative-risk frameworks,^78^ adolescents with multiple symptoms exhibited greater interpersonal impairment than those with single-year or single-symptom exposure. These patterns suggest that repeated or multifaceted symptom presentations impose both increasing psychological demands, as well as interpersonal challenges for adolescents. Associations with social withdrawal showed the clearest strengthening across study years, whereas peer victimization remained relatively stable across the years in which it was assessed. Family conflict showed some increase in point estimates over time, but the joint interaction tests did not survive FDR correction. The stability of victimization differences across assessment waves may reflect not only the structural persistence of peer contexts across adolescence but also the possibility that adolescents with eating disorder symptoms develop a heightened sensitivity to social threat that shapes how peer interactions are perceived and remembered over time.^98^^,^^99^ Additionally, school connectedness showed later-emerging differences. This could reflect the increasing salience of institutional belonging as adolescents assume greater autonomy within academic settings.^100–102^

The symptom-specific analyses clarify an important interpretive point about what “symptom exposure” represents in this cohort. Although any symptom histories were relatively common, the interpersonal correlates were not evenly distributed across the 5 KSADS symptoms. Across outcomes, the most consistent symptom correlates of poorer social functioning were binge eating and fear of becoming fat, whereas emaciation/thinness showed limited adjusted associations, and vomiting showed selective associations despite low prevalence. This suggests that interpersonal correlates of disordered eating are symptom-selective, rather than a diffuse consequence of any single symptom.

Binge eating merits further attention because it was both relatively common, compared to other symptoms in this cohort, and was the symptom with the clearest social health consequences. The interpersonal model of binge eating^103^ typically specifies a proximal pathway in which interpersonal challenges contribute to elevations in negative affect that then increase risk for binge eating as a coping response,^37^^,^^104^ and evidence on binge eating disorder treatment is consistent with this mechanism insofar as reductions in interpersonal problems covary with reductions in binge eating episodes through reductions in negative affect.^38^ At the same time, the present longitudinal results suggest that binge eating may be prospectively associated with poorer later interpersonal functioning. Thus, binge episodes can plausibly introduce secrecy or avoidance that alters day-to-day peer and family interactions, thereby generating additional interpersonal stressors that feed back into negative coping states and reinforce binge eating over time.^105–107^ This reciprocal framing also fits ecological momentary assessment (EMA) research that reports binge eating is tied to short-lived increases in negative affect and that interpersonal functioning shapes when affect becomes binge-triggering,^108^ implying that adolescent social health may operate both as an antecedent context for binge episodes and as a downstream consequence once binge eating emerges. Notably, this momentary evidence also suggests that between-person differences in average distress may be less informative than within-person deviations from one’s own baseline. In other words, binge eating may be triggered by mood changes that unfold over hours, whereas the social consequences of repeated episodes are more likely to persist long enough to be detectable in year-to-year change.

Two aspects of the binge-eating social health pattern were especially prominent in our results: social withdrawal and peer victimization. Peer victimization is both socially evaluative and difficult to escape in adolescence.^109^ Social exclusion can intensify negative arousal and self-focused distress,^110^ and binge eating may function as an immediately accessible strategy for downregulating that state when other coping resources are limited.^111^ Social withdrawal, in contrast, is less easily interpreted as a discrete “trigger” of binge eating and is better understood as a participation-level shift that can both follow from social threat and help maintain symptoms once binge eating is present. Withdrawal reduces exposure to supportive peer interactions and opportunities for repair, increases time spent in isolation, and leaves fewer interpersonal routes for affect to recover,^41^ such that eating may become a more prominent strategy for managing distress in the absence of those interpersonal resources, thereby reinforcing the cycle over time. This is consistent with evidence that social avoidance is a clinically meaningful interpersonal feature in binge eating presentations and can be linked to poorer outcomes,^112^ and with EMA findings showing that interpersonal style shapes how strongly negative affect translates into binge risk.^108^ These patterns suggest that social withdrawal may be a particularly consequential feature of binge eating in adolescence because it gradually narrows the relational options through which distress might otherwise be resolved.

Two findings diverged from expectation. First, prosocial behavior was unrelated to eating disorder symptoms after adjustment for covariates. This null finding indicates that adolescents exhibiting disordered eating behaviors did not differ from peers in their tendency to engage in supportive or helpful behaviors. Interpersonal functioning differences may therefore manifest more through reduced participation rather than diminished empathy or prosocial motivation. This inference is reinforced by the symptom-specific models, in which no symptom terms survived correction for prosocial behavior.

These findings extend developmental and behavioral medicine perspectives by framing disordered eating as a social health phenomenon.^25^ The consistent associations with social withdrawal and family conflict suggest that disordered eating co-occurs with interpersonal difficulties and, in lagged models, is prospectively associated with later differences in several social outcomes.^39^^,^^113^ In this sense, symptom exposure appears to relate to how adolescents participate in everyday relationships, not simply how they feel about them. Additionally, the diverging results between prosocial motivation and social participation clarify an important theoretical distinction. Adolescents exhibiting disordered eating behaviors did not differ from peers in prosocial tendencies. Instead, the challenge appears to involve the deployment of affiliation through reduced participation (eg, observed social withdrawal) in social contexts despite preserved intent. This distinction helps explain why relational disengagement may persist even when empathetic concern or helping behavior remains typical.

Several mechanisms could account for these interpersonal disruptions. Avoidant behavior, for instance, may elicit fewer invitations to social events or opportunities to reengage. Differences in school connectedness that emerged later in adolescence suggest that transitions, such as entry into secondary school, may represent sensitive periods for intervention. Supporting belonging during these transitions could prevent the stabilization of withdrawal and conflict into enduring social patterns. Future research should test potential mediators linking disordered eating to interpersonal functioning, including social anxiety,^45^^,^^114^ interaction fatigue^115–117^ (eg, social energy), and perceived restriction of autonomy.^118–120^ Identifying when and for whom these processes operate can inform the design of family- and school-based interventions that maintain social engagement even in the presence of ongoing symptoms.

In lagged autoregressive models that adjusted for the prior level of each social outcome, eating disorder symptoms at the prior wave predicted small but reliable changes in several outcomes at the subsequent wave, including withdrawal, family conflict, relational and reputational victimization, and school functioning. This prospective pattern is consistent with symptom-linked worsening in social functioning over time, even while it remains plausible that reciprocal processes also operate. Relatedly, results were broadly robust to expanded covariate adjustment: when time-aligned visit age, pubertal development, and same-wave internalizing symptoms were added (and withdrawal was excluded by design), associations with peer victimization and family conflict remained evident, and links with school climate and involvement persisted for the ≥1 symptom exposure. This pattern suggests that the primary findings are not fully explained by same-wave internalizing symptoms or pubertal development, although shared causes and time-varying confounding remain important considerations.

### Strengths and limitations

Strengths of this study include the large, demographically diverse cohort, annual repeated assessments, and the integration of multiple relational contexts. Additionally, the use of population weighting and cluster-robust variance estimators enhances external validity. Limitations include reliance on questionnaire-based symptoms rather than clinical diagnoses, which may conflate heterogeneous behaviors and levels of severity. Despite the longitudinal design, directionality remains limited because unmeasured variables (eg, sleep disturbance, pubertal development not captured by the available measure, neurodevelopmental traits, or time-varying internalizing symptoms) could contribute to both disordered eating and social impairment.

Because parents reported both eating disorder symptoms and social withdrawal, some degree of shared-reporter bias is possible, which could inflate the observed association between these constructs. However, parent report should not be treated as an interchangeable proxy for participant report, particularly as they enter adolescence. In a community cohort, adolescents endorsed substantially higher rates of disordered eating symptoms than their parents, with especially large discrepancies for bulimic behaviors such as purging and a general pattern of parent under-endorsement across symptoms.^84^ In a treatment-seeking clinical sample assessed via interview, parent-youth concordance was only poor to moderate overall, with notably weak agreement for symptom frequency and for internal cognitive features of eating concerns, and with adolescents tending to report higher levels of eating-disorder cognitions than parents.^121^ These findings suggest that caregiver-only symptom ascertainment likely under-captures less observable cognitions (eg, fear of weight gain) and more concealed behaviors (eg, vomiting), and that our exposure measure is best interpreted as caregiver-detected symptoms rather than the full distribution of adolescent disordered eating.

This has 2 concrete implications for the present findings. First, under-ascertainment would be expected to bias associations toward the null to the extent that adolescents with symptoms not detected by caregivers are misclassified as unexposed; in other words, the observed associations with adolescent-reported peer, family, and school functioning may be conservative estimates of the associations that would be obtained with adolescent symptom report.^84^^,^^121^ Second, because caregiver detection is more likely when symptoms are visible, severe, persistent, or accompanied by broader impairment, our estimates may be most representative of adolescents whose eating-related symptoms have already become noticeable within the family context. This limits generalizability to adolescents with more surreptitious or undisclosed symptoms, and it also means that the observed links with social health may partly reflect a subset of families in which symptoms are detectable and likely embedded in wider patterns of distress.

Information on evaluation or treatment exposure (eg, referral, family-based therapy, psychotherapy, or medication) and treatment response was not available. As a result, potential differences between treated and untreated adolescents in social functioning cannot be ruled out, and treatment initiated for more severe presentations could contribute to observed associations between symptom severity and social health. Effects were modest in size but observable across contexts and were strongest when symptoms were recurrent or complex. Accordingly, estimates should be interpreted as average population differences rather than clinical effects.

Together, these findings position adolescent disordered eating as a pattern that erodes participation across everyday interpersonal contexts, warranting recognition of social health as a measurable health outcome alongside physical and mental health. Routine assessment of social health in clinical and school settings could expose interpersonal costs early and offer points of intervention before withdrawal and conflict stabilize. Future work should test mediators, symptom onset timing, and developmental timing using designs that separate within-person change from stable traits and employ richer characterization of eating behaviors.

## Supplementary Material

kaag029_Supplementary_Data

## Data Availability

Data are available to qualified researchers through the ABCD Study data access process and the NIH National Data Archive (NDA), subject to approvals and data use agreements.

## Author Biographies

Christopher D. Otmar, PhD https://orcid.org/0000-0001-5433-1612 is a Postdoctoral Fellow in the Department of Pediatrics at University of California, San Francisco.

Colbey Ricklefs, MD, MPH https://orcid.org/0009-0009-6214-8557 is a Clinical Fellow in the Department of Pediatrics and a Postdoctoral Research Fellow in the Center for AIDS Prevention Studies at University of California, San Francisco.

Jason M. Nagata, MD https://orcid.org/0000-0002-6541-0604 is an Associate Professor in the Department of Pediatrics at University of California, San Francisco.

## Study registration

This is a secondary observational analysis without an intervention trial.

*Analytic plan registration*: Not preregistered.

## Analytic code availability

Available from the authors upon reasonable request.

## Materials availability

Not applicable; no novel instruments or lab materials were used beyond standard questionnaires.

*Reporting standards*: The study followed STROBE guidelines for observational cohort studies, with adherence described in the Methods.
