# Supplementary material for: Disordered eating behavior and adolescent social health: evidence of interpersonal disruption across time and relationships
Source: Ann Behav Med. 2026 Jun 16;60(1):kaag029. doi: 10.1093/abm/kaag029 (PMC13271245; doi:10.1093/abm/kaag029)
Supplement: kaag029_Supplementary_Data [file kaag029_supplementary_data.docx]

| **Table S1** | | | | | |
| --- | --- | --- | --- | --- | --- |
| *Outcome Descriptives and Missingness by Year* | | | | | |
| **Outcome** | **Year** | ***n*** | **Mean** | **SD** | **% missing** |
| Withdrawal (CBCL) | 0 | 11,861 | 0.13 | 0.23 | 0.1% |
|  | 1 | 11,201 | 0.14 | 0.24 | 0.2% |
|  | 2 | 10,897 | 0.15 | 0.26 | 0.7% |
|  | 3 | 10,197 | 0.17 | 0.28 | 2.4% |
|  | 4 | 9,494 | 0.19 | 0.30 | 2.4% |
| Prosocial behavior (SDQ) | 0 | 11,835 | 1.68 | 0.37 | 0.3% |
|  | 1 | 11,204 | 1.71 | 0.34 | 0.1% |
|  | 2 | 10,937 | 1.70 | 0.37 | 0.3% |
|  | 3 | 10,438 | 1.69 | 0.36 | 0.1% |
|  | 4 | 9,717 | 1.66 | 0.38 | 0.1% |
| Overt victimization (PEQ) | 0 | — | — | — | 100.0% |
|  | 1 | — | — | — | 100.0% |
|  | 2 | 10,946 | 3.67 | 1.37 | 0.2% |
|  | 3 | 10,420 | 3.52 | 1.17 | 0.2% |
|  | 4 | 9,658 | 3.52 | 1.18 | 0.8% |
| Relational victimization (PEQ) | 0 | — | — | — | 100.0% |
|  | 1 | — | — | — | 100.0% |
|  | 2 | 10,946 | 4.73 | 1.92 | 0.2% |
|  | 3 | 10,420 | 4.57 | 1.86 | 0.2% |
|  | 4 | 9,658 | 4.59 | 1.92 | 0.8% |
| Reputational victimization (PEQ) | 0 | — | — | — | 100.0% |
|  | 1 | — | — | — | 100.0% |
|  | 2 | 10,946 | 3.97 | 1.80 | 0.2% |
|  | 3 | 10,420 | 3.88 | 1.70 | 0.2% |
|  | 4 | 9,658 | 3.98 | 1.81 | 0.8% |
| Family conflict (FES) | 0 | 11,843 | 0.23 | 0.22 | 0.2% |
|  | 1 | 11,211 | 0.21 | 0.21 | 0.1% |
|  | 2 | 10,933 | 0.21 | 0.20 | 0.4% |
|  | 3 | 10,433 | 0.23 | 0.22 | 0.1% |
|  | 4 | 9,713 | 0.25 | 0.23 | 0.2% |
| School disengagement (SRPF) | 0 | 11,843 | 1.87 | 0.73 | 0.2% |
|  | 1 | 11,212 | 1.91 | 0.69 | 0.1% |
|  | 2 | 10,934 | 2.00 | 0.67 | 0.4% |
|  | 3 | 10,432 | 2.12 | 0.67 | 0.1% |
|  | 4 | 9,711 | 2.28 | 0.67 | 0.2% |
| School environment (SRPF) | 0 | 11,843 | 3.32 | 0.47 | 0.2% |
|  | 1 | 11,213 | 3.40 | 0.45 | 0.1% |
|  | 2 | 10,934 | 3.27 | 0.46 | 0.4% |
|  | 3 | 10,432 | 3.24 | 0.46 | 0.1% |
|  | 4 | 9,711 | 3.14 | 0.47 | 0.2% |
| School involvement (SRPF) | 0 | 11,842 | 3.26 | 0.59 | 0.2% |
|  | 1 | 11,213 | 3.32 | 0.57 | 0.1% |
|  | 2 | 10,934 | 3.16 | 0.59 | 0.4% |
|  | 3 | 10,432 | 3.11 | 0.57 | 0.1% |
|  | 4 | 9,710 | 3.01 | 0.58 | 0.2% |
| *Note.* Means and standard deviations are calculated using nonmissing data at each assessment wave. Dashes indicate that the outcome was not assessed in that year. | | | | | |

| **Table S2** | | | |
| --- | --- | --- | --- |
| *Internal Consistency Reliability by Year* | | | |
| **Scale** | **Year** | ***n* complete** | **Cronbach’s α** |
| Withdrawal (CBCL) | 0 | 11,861 | 0.638 |
|  | 1 | 11,201 | 0.626 |
|  | 2 | 10,897 | 0.674 |
|  | 3 | 10,197 | 0.694 |
|  | 4 | 9,493 | 0.719 |
| Prosocial behavior (SDQ) | 0 | 11,835 | 0.583 |
|  | 1 | 11,205 | 0.525 |
|  | 2 | 10,937 | 0.654 |
|  | 3 | 10,438 | 0.627 |
|  | 4 | 9,717 | 0.639 |
| Family conflict (FES) | 0 | 11,841 | 0.677 |
|  | 1 | 11,206 | 0.669 |
|  | 2 | 10,933 | 0.645 |
|  | 3 | 10,433 | 0.682 |
|  | 4 | 9,713 | 0.717 |
| Overt victimization (PEQ) | 0 | — | — |
|  | 1 | — | — |
|  | 2 | 10,946 | 0.710 |
|  | 3 | 10,420 | 0.705 |
|  | 4 | 9,658 | 0.726 |
| Relational victimization (PEQ) | 0 | — | — |
|  | 1 | — | — |
|  | 2 | 10,946 | 0.710 |
|  | 3 | 10,420 | 0.727 |
|  | 4 | 9,658 | 0.758 |
| Reputational victimization (PEQ) | 0 | — | — |
|  | 1 | — | — |
|  | 2 | 10,946 | 0.811 |
|  | 3 | 10,420 | 0.821 |
|  | 4 | 9,658 | 0.838 |
| School disengagement (SRPF) | 0 | 11,843 | 0.200 |
|  | 1 | 11,212 | 0.189 |
|  | 2 | 10,934 | 0.264 |
|  | 3 | 10,432 | 0.314 |
|  | 4 | 9,711 | 0.290 |
| School environment (SRPF) | 0 | 11,841 | 0.608 |
|  | 1 | 11,213 | 0.650 |
|  | 2 | 10,934 | 0.691 |
|  | 3 | 10,432 | 0.698 |
|  | 4 | 9,711 | 0.706 |
| School involvement (SRPF) | 0 | 11,842 | 0.649 |
|  | 1 | 11,213 | 0.686 |
|  | 2 | 10,934 | 0.716 |
|  | 3 | 10,432 | 0.708 |
|  | 4 | 9,710 | 0.708 |
| *Note.* Cronbach’s α values are reported for participants with complete item data at each assessment wave. Dashes indicate that the scale was not administered in that year. Item counts were as follows: CBCL Withdrawal (5 items), Prosocial behavior (SDQ; 3 items), Family Conflict (FES; 9 items), Overt/Relational/Reputational Victimization (PEQ; 3 items each), School Disengagement (SRPF; 2 items), School Environment (SRPF; 6 items), and School Involvement (SRPF; 4 items). | | | |

| **Table S3** | |
| --- | --- |
| *Symptom Prevalence (Ever)* | |
| **Symptom** | ***n* (%)** |
| Binge eating | 1,762 (14.8) |
| Emaciation/thinness | 2,006 (16.9) |
| Fear of becoming fat | 591 (5.0) |
| Other weight control behaviors | 1,643 (13.8) |
| Vomiting for weight control | 261 (2.2) |
| *Note.* Percentages reflect the proportion of participants who ever endorsed each symptom (*N* = 11,868). | |

| **Table S4** | | | | | |
| --- | --- | --- | --- | --- | --- |
| *Symptom Prevalence by Year* | | | | | |
| **Symptom** | **Year 0 (%)** | **Year 1 (%)** | **Year 2 (%)** | **Year 3 (%)** | **Year 4 (%)** |
| Binge eating | 5.1 | 7.2 | 6.3 | 4.0 | 4.8 |
| Emaciation/thinness | 9.0 | 6.8 | 5.5 | 1.1 | 3.0 |
| Fear of becoming fat | 0.7 | 0.8 | 1.1 | 2.5 | 2.8 |
| Other weight control behaviors | 2.8 | 3.9 | 4.1 | 5.3 | 6.4 |
| Vomiting for weight control | 0.4 | 0.5 | 0.6 | 0.6 | 0.7 |
| *Note.* Percentages reflect the proportion of participants endorsing each symptom at each assessment wave. Year-specific sample sizes were: Year 0 (*N* = 11,737), Year 1 (*N* = 11,089), Year 2 (*N* = 10,729), Year 3 (*N* = 9,950), and Year 4 (*N* = 8,348). | | | | | |

| **Table S5** | | | | | |
| --- | --- | --- | --- | --- | --- |
| *Joint Tests of Time-Varying Associations Between ED Symptoms and Social Outcomes (FDR-Adjusted)* | | | | | |
| **Outcome** | **ED symptom contrast** | **Wald χ²** | **df** | ***p*** | ***q*** |
| Withdrawal (CBCL) | ≥1 symptom | 18.1 | 4 | 0.001 | 0.009 |
|  | ≥1 symptom (proportion) | 13.5 | 4 | 0.009 | 0.054 |
|  | ≥2 symptoms | 2.17 | 4 | 0.705 | 0.846 |
|  | ≥2 symptoms (proportion) | 1.47 | 4 | 0.832 | 0.928 |
| Prosocial behavior (SDQ) | ≥1 symptom | 12.1 | 4 | 0.016 | 0.072 |
|  | ≥1 symptom (proportion) | 19.1 | 4 | 0.001 | 0.009 |
|  | ≥2 symptoms | 8.99 | 4 | 0.061 | 0.136 |
|  | ≥2 symptoms (proportion) | 13 | 4 | 0.011 | 0.058 |
| Overt victimization (PEQ) | ≥1 symptom | 1.02 | 2 | 0.601 | 0.746 |
|  | ≥1 symptom (proportion) | 2.22 | 2 | 0.329 | 0.493 |
|  | ≥2 symptoms | 0.03 | 2 | 0.987 | 0.987 |
|  | ≥2 symptoms (proportion) | 0.32 | 2 | 0.850 | 0.928 |
| Relational victimization (PEQ) | ≥1 symptom | 2.09 | 2 | 0.351 | 0.506 |
|  | ≥1 symptom (proportion) | 1.62 | 2 | 0.444 | 0.571 |
|  | ≥2 symptoms | 0.42 | 2 | 0.811 | 0.928 |
|  | ≥2 symptoms (proportion) | 0.15 | 2 | 0.927 | 0.953 |
| Reputational victimization (PEQ) | ≥1 symptom | 3.7 | 2 | 0.158 | 0.258 |
|  | ≥1 symptom (proportion) | 4.16 | 2 | 0.125 | 0.237 |
|  | ≥2 symptoms | 0.26 | 2 | 0.880 | 0.932 |
|  | ≥2 symptoms (proportion) | 1.88 | 2 | 0.392 | 0.522 |
| Family conflict (FES) | ≥1 symptom | 10.7 | 4 | 0.030 | 0.098 |
|  | ≥1 symptom (proportion) | 8.88 | 4 | 0.064 | 0.136 |
|  | ≥2 symptoms | 4.22 | 4 | 0.377 | 0.522 |
|  | ≥2 symptoms (proportion) | 5.33 | 4 | 0.255 | 0.400 |
| School disengagement (SRPF) | ≥1 symptom | 17.9 | 4 | 0.001 | 0.009 |
|  | ≥1 symptom (proportion) | 11.9 | 4 | 0.018 | 0.072 |
|  | ≥2 symptoms | 19.2 | 4 | 0.001 | 0.009 |
|  | ≥2 symptoms (proportion) | 22.2 | 4 | 0.001 | 0.007 |
| School environment (SRPF) | ≥1 symptom | 11.6 | 4 | 0.021 | 0.074 |
|  | ≥1 symptom (proportion) | 9.55 | 4 | 0.049 | 0.133 |
|  | ≥2 symptoms | 9.41 | 4 | 0.052 | 0.133 |
|  | ≥2 symptoms (proportion) | 6.84 | 4 | 0.145 | 0.258 |
| School involvement (SRPF) | ≥1 symptom | 10 | 4 | 0.040 | 0.121 |
|  | ≥1 symptom (proportion) | 8.96 | 4 | 0.062 | 0.136 |
|  | ≥2 symptoms | 6.65 | 4 | 0.156 | 0.258 |
|  | ≥2 symptoms (proportion) | 8.29 | 4 | 0.082 | 0.163 |
| *Note.* Wald χ² statistics test the joint significance of ED × year interaction terms from GEE models. *q* values reflect false discovery rate adjustment across outcomes. | | | | | |

| **Table S6** | | | | | | | | | | | | |
| --- | --- | --- | --- | --- | --- | --- | --- | --- | --- | --- | --- | --- |
| *Year-Specific Associations Between ED Symptoms and Social Outcomes (FDR-Adjusted)* | | | | | | | | | | | | |
| **Withdrawal (CBCL)** | | | | | |  | **Prosocial Behavior (SDQ)** | | | | | |
| **ED measure** | **Year** | **β** | **SE** | ***p*** | ***q*** |  | **ED measure** | **Year** | **β** | **SE** | ***p*** | ***q*** |
| ≥1 symptom | 0 | 0.207 | 0.021 | < .001 | < .001 |  | ≥1 symptom | 0 | 0.032 | 0.022 | 0.146 | 0.178 |
|  | 1 | 0.252 | 0.022 | < .001 | < .001 |  |  | 1 | −0.044 | 0.021 | 0.035 | 0.051 |
|  | 2 | 0.281 | 0.024 | < .001 | < .001 |  |  | 2 | −0.048 | 0.023 | 0.039 | 0.056 |
|  | 3 | 0.297 | 0.026 | < .001 | < .001 |  |  | 3 | −0.029 | 0.023 | 0.219 | 0.256 |
|  | 4 | 0.314 | 0.029 | < .001 | < .001 |  |  | 4 | −0.030 | 0.025 | 0.230 | 0.262 |
| ≥1 symptom (p) | 0 | 0.658 | 0.063 | < .001 | < .001 |  | ≥1 symptom (p) | 0 | 0.125 | 0.048 | 0.009 | 0.015 |
|  | 1 | 0.748 | 0.062 | < .001 | < .001 |  |  | 1 | −0.086 | 0.05 | 0.086 | 0.114 |
|  | 2 | 0.843 | 0.068 | < .001 | < .001 |  |  | 2 | −0.109 | 0.054 | 0.043 | 0.060 |
|  | 3 | 0.882 | 0.073 | < .001 | < .001 |  |  | 3 | −0.085 | 0.055 | 0.122 | 0.155 |
|  | 4 | 0.898 | 0.078 | < .001 | < .001 |  |  | 4 | −0.042 | 0.06 | 0.482 | 0.515 |
| ≥2 symptoms | 0 | 0.409 | 0.057 | < .001 | < .001 |  | ≥2 symptoms | 0 | 0.095 | 0.04 | 0.019 | 0.030 |
|  | 1 | 0.439 | 0.055 | < .001 | < .001 |  |  | 1 | 0.02 | 0.042 | 0.642 | 0.663 |
|  | 2 | 0.487 | 0.059 | < .001 | < .001 |  |  | 2 | −0.003 | 0.042 | 0.950 | 0.953 |
|  | 3 | 0.47 | 0.061 | < .001 | < .001 |  |  | 3 | −0.031 | 0.043 | 0.469 | 0.504 |
|  | 4 | 0.462 | 0.071 | < .001 | < .001 |  |  | 4 | −0.067 | 0.05 | 0.185 | 0.218 |
| ≥2 symptoms (p) | 0 | 1.43 | 0.181 | < .001 | < .001 |  | ≥2 symptoms (p) | 0 | 0.294 | 0.116 | 0.011 | 0.018 |
|  | 1 | 1.42 | 0.176 | < .001 | < .001 |  |  | 1 | −0.020 | 0.131 | 0.878 | 0.889 |
|  | 2 | 1.6 | 0.19 | < .001 | < .001 |  |  | 2 | −0.156 | 0.139 | 0.263 | 0.297 |
|  | 3 | 1.7 | 0.236 | < .001 | < .001 |  |  | 3 | −0.167 | 0.136 | 0.220 | 0.256 |
|  | 4 | 1.52 | 0.226 | < .001 | < .001 |  |  | 4 | −0.215 | 0.178 | 0.227 | 0.261 |
| **Overt Victimization (PEQ)** | | | | | |  | **Relational Victimization (PEQ)** | | | | | |
| **ED measure** | **Year** | **β** | **SE** | ***p*** | ***q*** |  | **ED measure** | **Year** | **β** | **SE** | ***p*** | ***q*** |
| ≥1 symptom | 2 | 0.099 | 0.025 | < .001 | < .001 |  | ≥1 symptom | 2 | 0.12 | 0.024 | < .001 | < .001 |
|  | 3 | 0.075 | 0.022 | < .001 | < .001 |  |  | 3 | 0.089 | 0.023 | < .001 | < .001 |
|  | 4 | 0.076 | 0.023 | < .001 | 0.002 |  |  | 4 | 0.085 | 0.024 | < .001 | < .001 |
| ≥1 symptom (p) | 2 | 0.323 | 0.063 | < .001 | < .001 |  | ≥1 symptom (p) | 2 | 0.337 | 0.063 | < .001 | < .001 |
|  | 3 | 0.256 | 0.055 | < .001 | < .001 |  |  | 3 | 0.275 | 0.058 | < .001 | < .001 |
|  | 4 | 0.216 | 0.056 | < .001 | < .001 |  |  | 4 | 0.248 | 0.062 | < .001 | < .001 |
| ≥2 symptoms | 2 | 0.166 | 0.055 | 0.002 | 0.004 |  | ≥2 symptoms | 2 | 0.16 | 0.051 | < .001 | 0.003 |
|  | 3 | 0.173 | 0.045 | < .001 | < .001 |  |  | 3 | 0.184 | 0.047 | < .001 | < .001 |
|  | 4 | 0.165 | 0.053 | 0.002 | 0.003 |  |  | 4 | 0.194 | 0.052 | < .001 | < .001 |
| ≥2 symptoms (p) | 2 | 0.645 | 0.178 | < .001 | < .001 |  | ≥2 symptoms (p) | 2 | 0.613 | 0.185 | < .001 | 0.002 |
|  | 3 | 0.665 | 0.163 | < .001 | < .001 |  |  | 3 | 0.674 | 0.161 | < .001 | < .001 |
|  | 4 | 0.56 | 0.167 | < .001 | < .001 |  |  | 4 | 0.621 | 0.18 | < .001 | < .001 |
| **Reputational Victimization (PEQ)** | | | | | |  | **Family Conflict (FES)** | | | | | |
| **ED measure** | **Year** | **β** | **SE** | ***p*** | ***q*** |  | **ED measure** | **Year** | **β** | **SE** | ***p*** | ***q*** |
| ≥1 symptom | 2 | 0.147 | 0.024 | < .001 | < .001 |  | ≥1 symptom | 0 | 0.076 | 0.022 | 0.001 | 0.001 |
|  | 3 | 0.106 | 0.024 | < .001 | < .001 |  |  | 1 | 0.09 | 0.022 | 0.001 | 0.001 |
|  | 4 | 0.101 | 0.026 | < .001 | < .001 |  |  | 2 | 0.117 | 0.022 | 0.001 | 0.001 |
| ≥1 symptom (p) | 2 | 0.433 | 0.064 | < .001 | < .001 |  |  | 3 | 0.14 | 0.024 | 0.001 | 0.001 |
|  | 3 | 0.36 | 0.062 | < .001 | < .001 |  |  | 4 | 0.164 | 0.026 | 0.001 | 0.001 |
|  | 4 | 0.285 | 0.068 | < .001 | < .001 |  | ≥1 symptom (p) | 0 | 0.189 | 0.052 | 0.001 | 0.001 |
| ≥2 symptoms | 2 | 0.259 | 0.059 | < .001 | < .001 |  |  | 1 | 0.234 | 0.052 | 0.001 | 0.001 |
|  | 3 | 0.265 | 0.054 | < .001 | < .001 |  |  | 2 | 0.249 | 0.051 | 0.001 | 0.001 |
|  | 4 | 0.234 | 0.059 | < .001 | < .001 |  |  | 3 | 0.352 | 0.058 | 0.001 | 0.001 |
| ≥2 symptoms (p) | 2 | 0.937 | 0.208 | < .001 | < .001 |  |  | 4 | 0.36 | 0.062 | 0.001 | 0.001 |
|  | 3 | 0.992 | 0.193 | < .001 | < .001 |  | ≥2 symptoms | 0 | 0.09 | 0.045 | 0.048 | 0.065 |
|  | 4 | 0.728 | 0.198 | < .001 | < .001 |  |  | 1 | 0.097 | 0.045 | 0.031 | 0.046 |
|  |  |  |  |  |  |  |  | 2 | 0.113 | 0.043 | 0.009 | 0.015 |
|  |  |  |  |  |  |  |  | 3 | 0.176 | 0.05 | 0.001 | 0.001 |
|  |  |  |  |  |  |  |  | 4 | 0.182 | 0.054 | 0.001 | 0.001 |
|  |  |  |  |  |  |  | ≥2 symptoms (p) | 0 | 0.305 | 0.132 | 0.021 | 0.032 |
|  |  |  |  |  |  |  |  | 1 | 0.287 | 0.138 | 0.038 | 0.056 |
|  |  |  |  |  |  |  |  | 2 | 0.293 | 0.136 | 0.031 | 0.047 |
|  |  |  |  |  |  |  |  | 3 | 0.585 | 0.161 | 0.001 | 0.001 |
|  |  |  |  |  |  |  |  | 4 | 0.501 | 0.166 | 0.003 | 0.005 |
| **School Disengagement (SRPF)** | | | | | |  | **School Environment (SRPF)** | | | | | |
| **ED measure** | **Year** | **β** | **SE** | ***p*** | ***q*** |  | **ED measure** | **Year** | **β** | **SE** | ***p*** | ***q*** |
| ≥1 symptom | 0 | −0.035 | 0.023 | 0.131 | 0.165 |  | ≥1 symptom | 0 | −0.045 | 0.023 | 0.044 | 0.061 |
|  | 1 | 0.044 | 0.022 | 0.046 | 0.064 |  |  | 1 | −0.090 | 0.022 | 0.001 | 0.001 |
|  | 2 | 0.056 | 0.022 | 0.011 | 0.018 |  |  | 2 | −0.095 | 0.023 | 0.001 | 0.001 |
|  | 3 | 0.077 | 0.022 | 0.001 | 0.001 |  |  | 3 | −0.133 | 0.023 | 0.001 | 0.001 |
|  | 4 | 0.053 | 0.022 | 0.02 | 0.031 |  |  | 4 | −0.074 | 0.024 | 0.002 | 0.004 |
| ≥1 symptom (p) | 0 | −0.052 | 0.053 | 0.324 | 0.356 |  | ≥1 symptom (p) | 0 | −0.085 | 0.054 | 0.117 | 0.152 |
|  | 1 | 0.131 | 0.053 | 0.013 | 0.022 |  |  | 1 | −0.188 | 0.05 | 0.001 | 0.001 |
|  | 2 | 0.121 | 0.052 | 0.02 | 0.031 |  |  | 2 | −0.205 | 0.055 | 0.001 | 0.001 |
|  | 3 | 0.152 | 0.052 | 0.004 | 0.007 |  |  | 3 | −0.288 | 0.055 | 0.001 | 0.001 |
|  | 4 | 0.081 | 0.055 | 0.145 | 0.178 |  |  | 4 | −0.167 | 0.06 | 0.006 | 0.010 |
| ≥2 symptoms | 0 | −0.162 | 0.043 | 0.001 | 0.001 |  | ≥2 symptoms | 0 | −0.037 | 0.045 | 0.410 | 0.444 |
|  | 1 | −0.003 | 0.042 | 0.953 | 0.953 |  |  | 1 | −0.070 | 0.043 | 0.101 | 0.132 |
|  | 2 | 0.012 | 0.042 | 0.783 | 0.798 |  |  | 2 | −0.046 | 0.046 | 0.320 | 0.355 |
|  | 3 | 0.069 | 0.045 | 0.121 | 0.155 |  |  | 3 | −0.185 | 0.049 | 0.001 | 0.001 |
|  | 4 | 0.017 | 0.044 | 0.695 | 0.714 |  |  | 4 | −0.108 | 0.05 | 0.030 | 0.046 |
| ≥2 symptoms (p) | 0 | −0.465 | 0.12 | 0.001 | 0.001 |  | ≥2 symptoms (p) | 0 | −0.143 | 0.139 | 0.304 | 0.339 |
|  | 1 | 0.074 | 0.134 | 0.583 | 0.608 |  |  | 1 | −0.279 | 0.137 | 0.042 | 0.060 |
|  | 2 | 0.167 | 0.137 | 0.224 | 0.258 |  |  | 2 | −0.101 | 0.149 | 0.499 | 0.529 |
|  | 3 | 0.209 | 0.144 | 0.144 | 0.178 |  |  | 3 | −0.501 | 0.165 | 0.002 | 0.004 |
|  | 4 | 0.134 | 0.155 | 0.387 | 0.422 |  |  | 4 | −0.336 | 0.175 | 0.055 | 0.073 |
| **School Involvement (SRPF)** | | | | | |  |  |  |  |  |  |  |
| **ED measure** | **Year** | **β** | **SE** | ***p*** | ***q*** |  |  |  |  |  |  |  |
| ≥1 symptom | 0 | −0.034 | 0.022 | 0.129 | 0.163 |  |  |  |  |  |  |  |
|  | 1 | −0.102 | 0.022 | 0.001 | 0.001 |  |  |  |  |  |  |  |
|  | 2 | −0.097 | 0.023 | 0.001 | 0.001 |  |  |  |  |  |  |  |
|  | 3 | −0.098 | 0.023 | 0.001 | 0.001 |  |  |  |  |  |  |  |
|  | 4 | −0.089 | 0.024 | 0.001 | 0.001 |  |  |  |  |  |  |  |
| ≥1 symptom (p) | 0 | −0.078 | 0.052 | 0.134 | 0.167 |  |  |  |  |  |  |  |
|  | 1 | −0.228 | 0.052 | 0.001 | 0.001 |  |  |  |  |  |  |  |
|  | 2 | −0.206 | 0.053 | 0.001 | 0.001 |  |  |  |  |  |  |  |
|  | 3 | −0.219 | 0.054 | 0.001 | 0.001 |  |  |  |  |  |  |  |
|  | 4 | −0.153 | 0.058 | 0.009 | 0.015 |  |  |  |  |  |  |  |
| ≥2 symptoms | 0 | 0.026 | 0.041 | 0.529 | 0.558 |  |  |  |  |  |  |  |
|  | 1 | −0.059 | 0.043 | 0.164 | 0.196 |  |  |  |  |  |  |  |
|  | 2 | −0.047 | 0.043 | 0.282 | 0.317 |  |  |  |  |  |  |  |
|  | 3 | −0.090 | 0.046 | 0.050 | 0.068 |  |  |  |  |  |  |  |
|  | 4 | −0.065 | 0.046 | 0.161 | 0.195 |  |  |  |  |  |  |  |
| ≥2 symptoms (p) | 0 | 0.063 | 0.116 | 0.585 | 0.608 |  |  |  |  |  |  |  |
|  | 1 | −0.280 | 0.136 | 0.040 | 0.058 |  |  |  |  |  |  |  |
|  | 2 | −0.197 | 0.141 | 0.163 | 0.195 |  |  |  |  |  |  |  |
|  | 3 | −0.269 | 0.158 | 0.088 | 0.116 |  |  |  |  |  |  |  |
|  | 4 | −0.307 | 0.155 | 0.048 | 0.065 |  |  |  |  |  |  |  |
| Note. Year-specific coefficients are estimated relative to the reference year (Year 0 for withdrawal, prosocial behavior, family conflict, and school outcomes; Year 2 for peer victimization outcomes). q values reflect false discovery rate adjustment. | | | | | | | | | | | | |

| **Table S7** | | | | |
| --- | --- | --- | --- | --- |
| *Symptom-Specific Associations Between ED Symptoms and Social Outcomes (FDR-Adjusted)* | | | | |
| **Withdrawal (CBCL)** | | | | |
| **Symptom** | **β** | **SE** | ***p*** | ***q*** |
| Binge eating | 0.203 | 0.028 | < .001 | < .001 |
| Emaciation/thinness | 0.009 | 0.02 | 0.638 | 0.653 |
| Fear of becoming fat | 0.364 | 0.057 | < .001 | < .001 |
| Other weight control behaviors | 0.133 | 0.029 | < .001 | < .001 |
| Vomiting for weight control | 0.147 | 0.084 | 0.079 | 0.137 |
| **Prosocial Behavior (SDQ)** | | | | |
| **Symptom** | **β** | **SE** | ***p*** | ***q*** |
| Binge eating | −0.012 | 0.024 | 0.63 | 0.653 |
| Emaciation/thinness | −0.014 | 0.021 | 0.517 | 0.568 |
| Fear of becoming fat | −0.042 | 0.043 | 0.319 | 0.43 |
| Other weight control behaviors | 0.013 | 0.024 | 0.594 | 0.636 |
| Vomiting for weight control | −0.106 | 0.072 | 0.139 | 0.201 |
| **Overt Victimization (PEQ)** | | | | |
| **Symptom** | **β** | **SE** | ***p*** | ***q*** |
| Binge eating | 0.205 | 0.04 | < .001 | < .001 |
| Emaciation/thinness | −0.062 | 0.04 | 0.124 | 0.186 |
| Fear of becoming fat | 0.243 | 0.065 | < .001 | < .001 |
| Other weight control behaviors | 0.141 | 0.035 | < .001 | < .001 |
| Vomiting for weight control | 0.199 | 0.102 | 0.05 | 0.098 |
| **Relational Victimization (PEQ)** | | | | |
| **Symptom** | **β** | **SE** | ***p*** | ***q*** |
| Binge eating | 0.134 | 0.037 | < .001 | < .001 |
| Emaciation/thinness | 0.002 | 0.039 | 0.949 | 0.949 |
| Fear of becoming fat | 0.25 | 0.067 | < .001 | < .001 |
| Other weight control behaviors | 0.112 | 0.035 | < .001 | 0.004 |
| Vomiting for weight control | 0.194 | 0.074 | 0.009 | 0.019 |
| **Reputational Victimization (PEQ)** | | | | |
| **Symptom** | **β** | **SE** | ***p*** | ***q*** |
| Binge eating | 0.211 | 0.04 | < .001 | < .001 |
| Emaciation/thinness | 0.028 | 0.037 | 0.449 | 0.524 |
| Fear of becoming fat | 0.315 | 0.082 | < .001 | < .001 |
| Other weight control behaviors | 0.152 | 0.038 | < .001 | < .001 |
| Vomiting for weight control | 0.084 | 0.08 | 0.294 | 0.413 |
| **Family Conflict (FES)** | | | | |
| **Symptom** | **β** | **SE** | ***p*** | ***q*** |
| Binge eating | 0.071 | 0.025 | 0.004 | 0.011 |
| Emaciation/thinness | 0.018 | 0.022 | 0.419 | 0.509 |
| Fear of becoming fat | 0.134 | 0.045 | 0.003 | 0.008 |
| Other weight control behaviors | 0.055 | 0.025 | 0.027 | 0.059 |
| Vomiting for weight control | 0.053 | 0.066 | 0.415 | 0.509 |
| **School Disengagement (SRPF)** | | | | |
| **Symptom** | **β** | **SE** | ***p*** | ***q*** |
| Binge eating | 0.038 | 0.023 | 0.106 | 0.164 |
| Emaciation/thinness | −0.016 | 0.022 | 0.454 | 0.524 |
| Fear of becoming fat | 0.12 | 0.046 | 0.009 | 0.019 |
| Other weight control behaviors | 0.023 | 0.024 | 0.345 | 0.444 |
| Vomiting for weight control | 0.115 | 0.071 | 0.106 | 0.164 |
| **School Environment (SRPF)** | | | | |
| **Symptom** | **β** | **SE** | ***p*** | ***q*** |
| Binge eating | −0.044 | 0.024 | 0.071 | 0.128 |
| Emaciation/thinness | 0.015 | 0.021 | 0.488 | 0.549 |
| Fear of becoming fat | −0.094 | 0.05 | 0.059 | 0.11 |
| Other weight control behaviors | −0.088 | 0.025 | < .001 | < .001 |
| Vomiting for weight control | −0.185 | 0.063 | 0.004 | 0.009 |
| **School Involvement (SRPF)** | | | | |
| **Symptom** | **β** | **SE** | ***p*** | ***q*** |
| Binge eating | −0.046 | 0.022 | 0.037 | 0.075 |
| Emaciation/thinness | 0.02 | 0.02 | 0.325 | 0.43 |
| Fear of becoming fat | −0.181 | 0.044 | < .001 | < .001 |
| Other weight control behaviors | −0.093 | 0.024 | < .001 | < .001 |
| Vomiting for weight control | −0.099 | 0.06 | 0.098 | 0.163 |
| ***Note.*** β coefficients are from symptom-specific GEE models adjusted for covariates described in the main text. All models used an AR(1) working correlation structure. q values reflect false discovery rate adjustment across symptoms within outcome. | | | | |

| **Table S8** | | | | |
| --- | --- | --- | --- | --- |
| *Lagged Associations Between ED Symptoms and Social Outcomes (FDR-Adjusted)* | | | | |
| **Withdrawal (CBCL)** | | | | |
| **Lagged predictor** | **β** | **SE** | ***p*** | ***q*** |
| ≥1 symptom (t−1) | 0.086 | 0.015 | < .001 | < .001 |
| ≥2 symptoms (t−1) | 0.044 | 0.048 | 0.352 | 0.488 |
| **Prosocial Behavior (SDQ)** | | | | |
| **Lagged predictor** | **β** | **SE** | ***p*** | ***q*** |
| ≥1 symptom (t−1) | −0.011 | 0.015 | 0.443 | 0.514 |
| ≥2 symptoms (t−1) | −0.018 | 0.039 | 0.655 | 0.693 |
| **Overt Victimization (PEQ)** | | | | |
| **Lagged predictor** | **β** | **SE** | ***p*** | ***q*** |
| ≥1 symptom (t−1) | 0.047 | 0.023 | 0.041 | 0.081 |
| ≥2 symptoms (t−1) | 0.042 | 0.053 | 0.434 | 0.514 |
| **Relational Victimization (PEQ)** | | | | |
| **Lagged predictor** | **β** | **SE** | ***p*** | ***q*** |
| ≥1 symptom (t−1) | 0.058 | 0.024 | 0.013 | 0.040 |
| ≥2 symptoms (t−1) | 0.042 | 0.057 | 0.457 | 0.514 |
| **Reputational Victimization (PEQ)** | | | | |
| **Lagged predictor** | **β** | **SE** | ***p*** | ***q*** |
| ≥1 symptom (t−1) | 0.060 | 0.025 | 0.016 | 0.040 |
| ≥2 symptoms (t−1) | 0.104 | 0.065 | 0.109 | 0.164 |
| **Family Conflict (FES)** | | | | |
| **Lagged predictor** | **β** | **SE** | ***p*** | ***q*** |
| ≥1 symptom (t−1) | 0.057 | 0.014 | < .001 | < .001 |
| ≥2 symptoms (t−1) | 0.100 | 0.040 | 0.013 | 0.040 |
| **School Disengagement (SRPF)** | | | | |
| **Lagged predictor** | **β** | **SE** | ***p*** | ***q*** |
| ≥1 symptom (t−1) | 0.032 | 0.014 | 0.020 | 0.045 |
| ≥2 symptoms (t−1) | 0.065 | 0.038 | 0.084 | 0.137 |
| **School Environment (SRPF)** | | | | |
| **Lagged predictor** | **β** | **SE** | ***p*** | ***q*** |
| ≥1 symptom (t−1) | −0.051 | 0.014 | < .001 | 0.002 |
| ≥2 symptoms (t−1) | −0.081 | 0.043 | 0.058 | 0.104 |
| **School Involvement (SRPF)** | | | | |
| **Lagged predictor** | **β** | **SE** | ***p*** | ***q*** |
| ≥1 symptom (t−1) | −0.034 | 0.013 | 0.012 | 0.040 |
| ≥2 symptoms (t−1) | −0.013 | 0.038 | 0.738 | 0.738 |
| ***Note.*** β coefficients are from lagged GEE models predicting outcomes at time t from ED symptom status at time t−1, controlling for the prior level of the same outcome at time t−1, and adjusting for covariates described in the main text. All models used an AR(1) working correlation structure. q values reflect false discovery rate adjustment across outcomes. | | | | |

| **Table S9** | | | | | |
| --- | --- | --- | --- | --- | --- |
| *Sensitivity Models Including Age, Puberty, and Internalizing Symptoms (FDR-Adjusted)* | | | | | |
| **Prosocial Behavior (SDQ)** | | | | | |
| **Predictor** |  | **β** | **SE** | ***p*** | ***q*** |
| **Covariates** | Internalizing (CBCL T-score) | −0.004 | 0.001 | < .001 | — |
|  | Puberty (PDS mean) | −0.035 | 0.011 | < .001 | — |
|  | Visit age | 0.006 | 0.012 | 0.62 | — |
| **ED measures** | ≥1 symptom | 0 | 0.016 | 0.982 | 0.982 |
|  | ≥2 symptoms | 0.034 | 0.03 | 0.255 | 0.327 |
|  | ≥1 symptom (proportion) | 0.03 | 0.037 | 0.418 | 0.515 |
|  | ≥2 symptoms (proportion) | 0.071 | 0.099 | 0.474 | 0.562 |
| **Overt Victimization (PEQ)** | | | | | |
| **Predictor** |  | **β** | **SE** | ***p*** | ***q*** |
| **Covariates** | Internalizing (CBCL T-score) | 0.009 | 0.001 | < .001 | — |
|  | Puberty (PDS mean) | 0.108 | 0.013 | < .001 | — |
|  | Visit age | −0.033 | 0.013 | 0.01 | — |
| **ED measures** | ≥1 symptom | 0.05 | 0.018 | 0.004 | 0.011 |
|  | ≥2 symptoms | 0.092 | 0.039 | 0.018 | 0.036 |
|  | ≥1 symptom (proportion) | 0.175 | 0.044 | < .001 | < .001 |
|  | ≥2 symptoms (proportion) | 0.385 | 0.131 | 0.003 | 0.009 |
| **Relational Victimization (PEQ)** | | | | | |
| **Predictor** |  | **β** | **SE** | ***p*** | ***q*** |
| **Covariates** | Internalizing (CBCL T-score) | 0.011 | 0.001 | < .001 | — |
|  | Puberty (PDS mean) | 0.07 | 0.013 | < .001 | — |
|  | Visit age | 0.006 | 0.013 | 0.662 | — |
| **ED measures** | ≥1 symptom | 0.052 | 0.018 | 0.004 | 0.01 |
|  | ≥2 symptoms | 0.082 | 0.04 | 0.038 | 0.067 |
|  | ≥1 symptom (proportion) | 0.157 | 0.047 | < .001 | 0.003 |
|  | ≥2 symptoms (proportion) | 0.324 | 0.147 | 0.027 | 0.052 |
| **Reputational Victimization (PEQ)** | | | | | |
| **Predictor** |  | **β** | **SE** | ***p*** | ***q*** |
| **Covariates** | Internalizing (CBCL T-score) | 0.011 | 0.001 | < .001 | — |
|  | Puberty (PDS mean) | 0.12 | 0.014 | < .001 | — |
|  | Visit age | 0.028 | 0.014 | 0.036 | — |
| **ED measures** | ≥1 symptom | 0.072 | 0.019 | < .001 | < .001 |
|  | ≥2 symptoms | 0.157 | 0.046 | < .001 | 0.003 |
|  | ≥1 symptom (proportion) | 0.236 | 0.05 | < .001 | < .001 |
|  | ≥2 symptoms (proportion) | 0.581 | 0.167 | < .001 | 0.003 |
| **Family Conflict (FES)** | | | | | |
| **Predictor** |  | **β** | **SE** | ***p*** | ***q*** |
| **Covariates** | Internalizing (CBCL T-score) | 0.007 | 0.001 | < .001 | — |
|  | Puberty (PDS mean) | 0.088 | 0.011 | < .001 | — |
|  | Visit age | −0.007 | 0.012 | 0.542 | — |
| **ED measures** | ≥1 symptom | 0.085 | 0.017 | < .001 | < .001 |
|  | ≥2 symptoms | 0.07 | 0.035 | 0.046 | 0.074 |
|  | ≥1 symptom (proportion) | 0.18 | 0.041 | < .001 | < .001 |
|  | ≥2 symptoms (proportion) | 0.161 | 0.104 | 0.121 | 0.184 |
| **School Disengagement (SRPF)** | | | | | |
| **Predictor** |  | **β** | **SE** | ***p*** | ***q*** |
| **Covariates** | Internalizing (CBCL T-score) | 0.007 | 0.001 | < .001 | — |
|  | Puberty (PDS mean) | 0.076 | 0.011 | < .001 | — |
|  | Visit age | 0.043 | 0.011 | < .001 | — |
| **ED measures** | ≥1 symptom | 0.008 | 0.015 | 0.589 | 0.649 |
|  | ≥2 symptoms | −0.088 | 0.029 | 0.002 | 0.007 |
|  | ≥1 symptom (proportion) | −0.008 | 0.036 | 0.818 | 0.844 |
|  | ≥2 symptoms (proportion) | −0.239 | 0.087 | 0.006 | 0.014 |
| **School Environment (SRPF)** | | | | | |
| **Predictor** |  | **β** | **SE** | ***p*** | ***q*** |
| **Covariates** | Internalizing (CBCL T-score) | −0.008 | 0.001 | < .001 | — |
|  | Puberty (PDS mean) | −0.060 | 0.011 | < .001 | — |
|  | Visit age | −0.024 | 0.011 | 0.036 | — |
| **ED measures** | ≥1 symptom | −0.050 | 0.016 | < .001 | 0.005 |
|  | ≥2 symptoms | −0.020 | 0.032 | 0.54 | 0.617 |
|  | ≥1 symptom (proportion) | −0.077 | 0.037 | 0.041 | 0.068 |
|  | ≥2 symptoms (proportion) | −0.043 | 0.1 | 0.668 | 0.713 |
| **School Involvement (SRPF)** | | | | | |
| **Predictor** |  | **β** | **SE** | ***p*** | ***q*** |
| **Covariates** | Internalizing (CBCL T-score) | −0.008 | 0.001 | < .001 | — |
|  | Puberty (PDS mean) | −0.062 | 0.01 | < .001 | — |
|  | Visit age | −0.039 | 0.011 | < .001 | — |
| **ED measures** | ≥1 symptom | −0.043 | 0.016 | 0.009 | 0.018 |
|  | ≥2 symptoms | 0.047 | 0.031 | 0.127 | 0.185 |
|  | ≥1 symptom (proportion) | −0.051 | 0.038 | 0.182 | 0.253 |
|  | ≥2 symptoms (proportion) | 0.111 | 0.097 | 0.255 | 0.327 |
| ***Note.*** Sensitivity models adjusted for age, pubertal development, and internalizing symptoms. Withdrawal (CBCL) was not modeled due to conceptual and statistical overlap with the internalizing covariate. *q* values reflect false discovery rate adjustment across ED measures within outcome. | | | | | |
